# Characterization of Transcriptional Complexity during Longissimus Muscle Development in Bovines Using High-Throughput Sequencing

**DOI:** 10.1371/journal.pone.0064356

**Published:** 2013-06-07

**Authors:** Hua He, Xiaolin Liu

**Affiliations:** College of Animal Science and Technology, Northwest A&F University, Shaanxi Key Laboratory of Molecular Biology for Agriculture, Yangling, Shaanxi, People's Republic of China; University of Rome, Italy

## Abstract

**Background:**

Beef cattle are among the most economically important animals in the world because they are farmed for their meat and leather. However, a lack of genetic information remains an obstacle to understanding the mechanisms behind the development of this animal. Analysis of the beef cattle transcriptome and its expression profile data are essential to extending the genetic information resources for this species and would support studies on this animal.

**Results:**

RNA sequencing of beef cattle was performed using the Illumina High-Seq2000 platform. A total of 25,605,140 and 26,214,800 reads were sequenced for embryonic and adult pooled samples, respectively. We identified 24,464–29,994 novel transcript units in two pooled samples. In addition, 8,533–10,144 genes showed evidence of alternative splicing, in agreement with the finding that alternative 3′ splicing is the most common type of alternative splicing event in cattle. We detected the expression levels of 16,174 genes, and 6,800 genes exhibited differential expression between the two pooled samples with a false discovery rate ≤0.001. Using GO enrichment and KEGG pathway analysis, multiple GO term and biological pathways were found to be significantly enriched for differentially expressed genes. In addition, we discovered that 30,618–31,334 putative single nucleotide polymorphisms were located in coding regions.

**Conclusions:**

We obtained a high-quality beef cattle reference transcriptome using a high throughput sequencing approach, thereby providing a valuable resource for better understanding the beef cattle genome. The transcriptome data will facilitate future functional studies on the beef cattle genome and can be applied to breeding programs for cattle and closely related mammals.

## Introduction

As an elite yellow cattle breed, Qinchuan beef cattle have had a long history of feeding and breeding in China. The practice of selecting good cattle to present to the master has been recorded as early as 800 BC [1]. Qinchuan cattle were mainly used as draft animals throughout history. When Zhangqian brought back alfalfa seeds from the West in 126 BC via the Silk Road, people began to plant alfalfa for cattle feed on the Guanzhong Plain, the main production area of Qinchuan beef cattle. This resulted in tremendous improvements in Qinchuan beef cattle, particularly in relation to its body size, work ability, and individual meat yield.

Genetic background and pre-birth development are known to affect the composition of bovine muscle tissue [2], [3]. Prenatal muscle development is therefore a promising area of gene discovery regarding the molecular events that determine adult muscle phenotype.

However, the complexity of the bovine transcriptome has not yet been fully elucidated. Novel, high-throughput, deep-sequencing technologies are affecting genomic research by providing new strategies to analyze the functional complexity of transcriptomes. The RNA sequencing (RNA-Seq) approach produces millions of short cDNA reads that are mapped to a reference genome to obtain a genome-scale transcriptional map, which consists of the transcriptional structure and the expression level of each gene [4]. The holistic view of the transcriptome and its organization provided by the RNA-Seq method reveals many novel transcribed regions, splice isoforms, and single nucleotide polymorphisms (SNPs) and allows refinement of gene structures [5]–[10]. Finally, RNA-Seq generates absolute rather than relative gene expression measurements, thereby providing greater insight and accuracy than do microarrays [11], [12].

In the current study, we have performed the first global analysis of the beef cattle transcriptome during muscle development using the Illumina RNA-Seq method. Although our main aim was to validate the RNA-Seq technology and to set up a pipeline that allows observation of the level of gene expression, new transcripts, splice variants, and SNPs, we report here a comprehensive analysis of transcriptome dynamics that may serve as a blueprint of the gene expression profiles that occur during muscle development.

## Results

### Deep Sequencing of Bovine Longissimus Muscle Transcriptomes

Using RNA-Seq, this study compared the transcriptomic landscapes of longissimus muscle from embryo at day 135 post fertilization (Emb135d) versus 30-month-old (30M) adult cattle. To accomplish this, two rounds of linear amplification of mRNA were used, ensuring that each individual produced enough RNA input for analysis. Amplified RNA from three embryos and three adult bovines, respectively, all with the same sire, was pooled and sequenced on the High-Seq2000 system at BGI-Shenzhen, China, resulting in approximately 2 billion pair-end reads of 100 bp in length. The data set was analyzed according to the BGI bioinformatics protocols for RNA-seq. Table 1 presents the overall results of aligning sequencing reads to the bovine reference genome (UMD Bos taurus 3.1 (UMD 3.1), produced by the University of Maryland) and genes.

**Table 1:** Summary of sequence read alignments to the UMD 3.1 reference genome and gene.

Category	30M sample		Emb135 sample	
	Reads number	Percentage	Reads number	Percentage
Total reads	26,214,800	100.00%	25,605,140	100.00%
Total base pairs	2,359,332,000	100.00%	2,304,462,600	100.00%
Total mapped reads	20,427,874	77.92%	20,255,767	79.11%
Read perfectly matched to the reference genome	14,364,201	54.79%	14,300,096	55.85%
Read with ≤3 bp mismatch to the reference genome	6,063,673	23.13%	5,955,671	23.26%
Reads uniquely matched to the reference genome	19,713,327	75.20%	18,733,163	73.16%
Reads matched to the reference genome with multi-positions	714,547	2.73%	1,522,604	5.95%
Total unmapped reads to the reference genome	5,786,926	22.08%	5,349,373	20.89%
Total mapped reads to annotated genes	20,500,464	78.20%	18,898,384	73.81%
Read perfectly matched to the annotated genes	15,500,019	59.13%	14,859,948	58.04%
Read with ≤3 bp mismatch to the annotated genes	5,000,445	19.07%	4,038,436	15.77%
Reads uniquely matched to the annotated genes	17,615,556	67.20%	16,364,686	63.91%
Reads matched to the annotated genes with multi-positions	2,884,908	11.00%	2,533,698	9.90%
Total unmapped reads to annotated genes	5,714,336	21.80%	6,706,756	26.19%

Total reads: total number of sequencing reads. Total base pairs: total number of base pairs. Total mapped reads: the reads that can aligned to reference sequence and the ratio of it. We not only classified statistic by mismatch number but also classified by uniqueness of alignment postion, following 4 ranks are result of classified statistic, perfect match: in total mapped reads, no mismatch; ≤3 bp mismatch: in total mapped reads, mismatch number less than 3 bp; unique match: in total mapped reads, reads aligned to only one postion; multi-position match: in total mapped reads, reads aligned to two or more places. Total unmapped reads: reads that don't aligned to reference sequence.

Sequencing reads were analyzed using SOAP2 software [13] by alignment with the UMD 3.1 reference genome. Of the total sequenced reads, 79.11% and 77.92% were mapped to the UMD 3.1 reference genome for Emb135 and 30M samples, respectively. Of these, 73.16% and 75.20%, respectively, were uniquely mapped to specific regions in the bovine genome, and 73.81% and 78.20% of reads corresponded to reference genes with 63.91% and 67.20% uniquely matched reads, respectively (Table 1). Unmapped and multi-position matched reads were excluded from further analyses (Table 1).

### Identification and Analysis of Novel Transcribed Units

**Figure 1:**
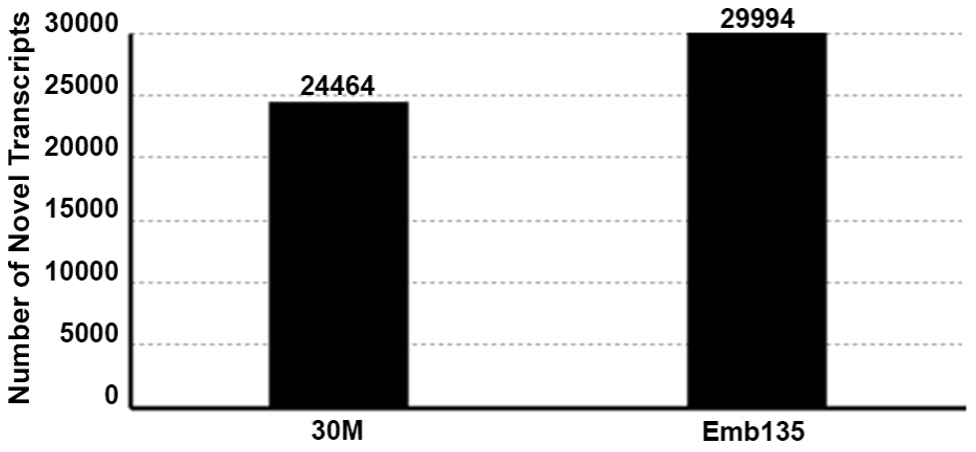
Statistics of novel transcripts. The abscissa shows the samples 30M and Emb135, and the ordinate represents the number of novel transcripts occurred in each sample.

Using the procedures described in the materials and methods section and those of Zhang et al. [14], we detected an extensive number of novel transcript units. The statistics of the novel transcripts are presented in Figure 1. In total, we obtained 29,994 and 24,464 novel transcript units in the pooled samples for Emb135 and 30M, respectively, with a mean length of 353 bp and with lengths ranging from 150 to 22,302 bp (Table S1). Many novel transcript units (28.54%–51.93%) have ≥1 exon, and the largest is 6,917 bp in length and contains 23 exons (Table S1).

### Alternative Splicing Events in the Bovine Transcriptome

**Figure 2:**
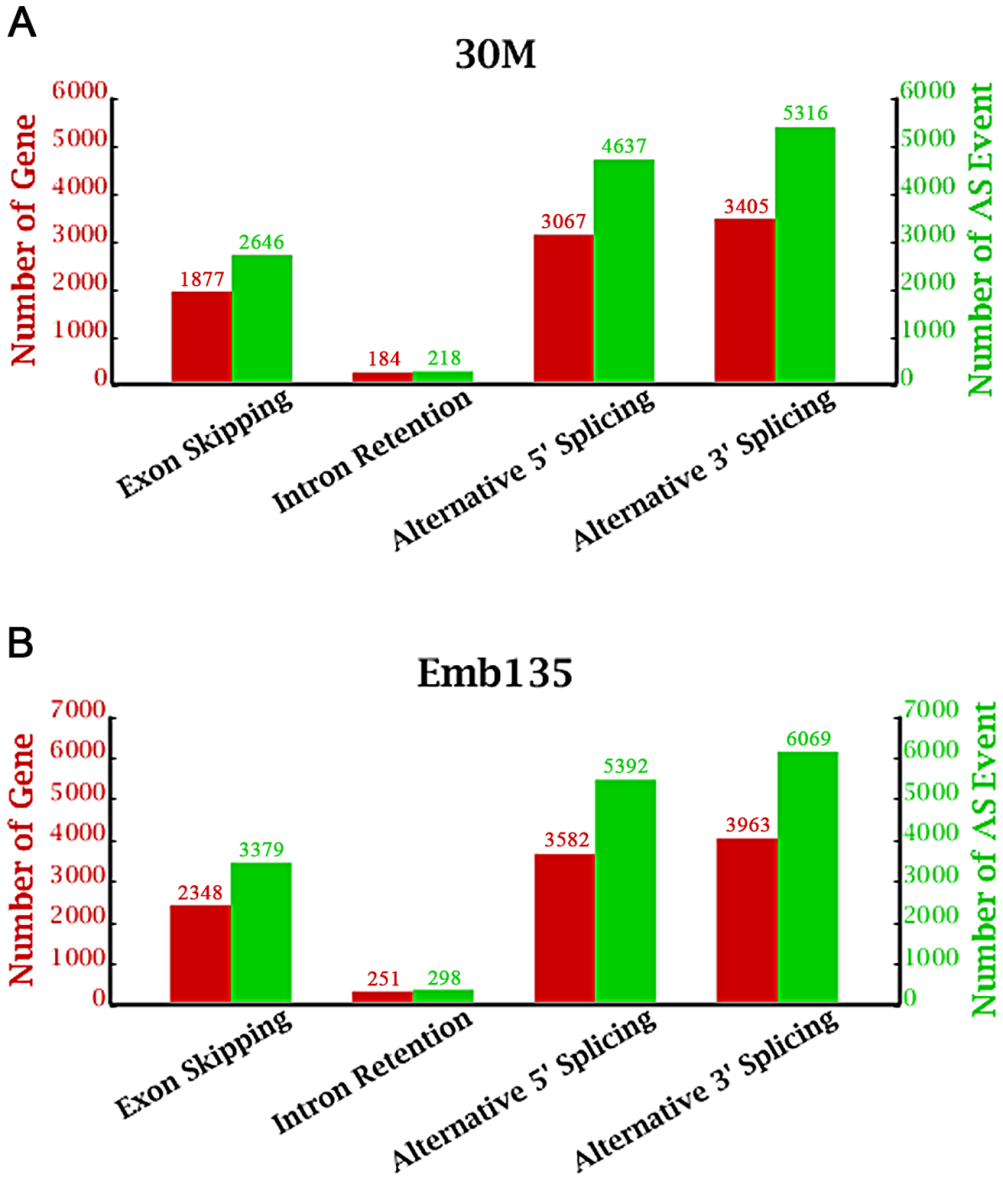
Statistics of alternative splicing events and genes in two samples. (A): The red bars show the distribution of the number of genes for each type of alternative splicing mode in the pooled 30M sample. The green bars indicate the number of alternative splicing events that occurred in each gene. (B) is same as A except that the results for the Emb135 sample are presented.

Alternative splicing (AS) plays a major role in the generation of proteomic and functional complexity in higher organisms [15], [16], [17], [18]. To explore potential AS events, we conducted computational analyses to determine all theoretical splicing junctions and then identified sequence reads that mapped to these regions. A brief introduction to the algorithms used to detect alternative splicing events follows: First, junction sites, which provide information about boundaries and the combinations of different exons in a transcript, are detected by SOAPsplice. Then, all junction sites of the same gene are used to distinguish the type of its alternative splicing event. Seven main types of alternative splicing exist: exon skipping, intron retention, and alternative 5′ splice site, alternative 3′ splice site, alternative first exon, alternative last exon, and mutually exclusive exon-splicing. The last three types are not included in our results due to the high rate of false positive results obtained when using the SOAPsplice software. Figure 2 (below) shows these alternative splicing events and the associated gene numbers that we determined through high throughput sequencing.

We found that approximately 66.6% of the alternatively spliced genes underwent multiple alternative splicing events, thus illustrating the complexity of the bovine transcriptome. Alternative 3′ splice site-splicing is the most common type of alternative splicing event, accounting for an average of 40.8% of all alternative splicing events in cattle; in contrast, intron retention is the least common type of splicing event, only constituting 1.7% in the pooled Emb135 sample and 2.0% in the pooled 30M sample. The average size of retained introns in the Emb135 sample is 354 bp (range: 64–3,168 bp). In the 30M sample, the average size of retained introns is 380 bp (range: 60–8,708 bp). Table S2 identifies AS events in each sample in detail.

### Refinement of Gene Structures

Gene structure was optimized according to the distribution of the reads, information from RNA-seq, and the annotation of the reference gene. First, we obtained the distribution of reads in the genome by aligning the continuous and overlapping reads to form a Transcription Active Region (TAR). Second, in accordance with the paired-end RNA-seq data, we connected the different TARs to form a potential gene model. Finally, we compared the gene model with the existing annotated gene to extend the gene's 5′- and 3′-ends. In the 30M pooled sample, more than 2,571 regions were extended at the 5′- end, and more than 4,625 regions had an extension at the 3′- end. In comparison, in the Emb135 pooled sample, at least 2,157 regions were extended at the 5′- end, and at least 3,531 regions had an extension at their 3′- end. Table S2 describes the 5′- and 3′- end extensions of genes in each sample.

### Identification of Differentially Expressed Genes

**Figure 3:**
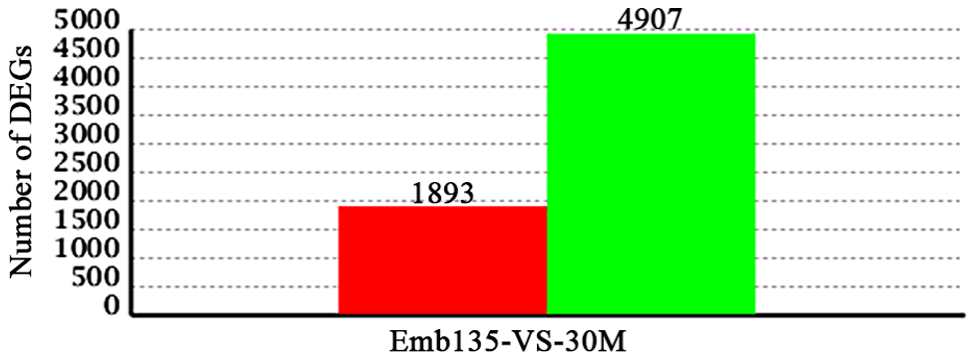
Statistics of differentially expressed genes (DEGs). Red and green bars above the X-axis denote genes with up- and down-regulated expression in Emb135-VS-30M samples, respectively. The Y-axis denote number of differentially expressed genes with up- and down-regulated expression in Emb135-VS-30M samples, respectively.

The RNA-Seq technique allows analysis of the differential expression profile via transcript abundance with a high sensitivity for transcripts expressed at low levels that would otherwise be undetectable using standard microarrays [19]. The differentially expressed genes were selected based on the expression profiles and the following criteria: (1) if the change in gene expression levels between Emb135 and 30M was greater than or equal to two-fold (|log_2_Ratio| ≥1) and (2) if the false discovery rate (FDR) was less than or equal to 0.001. In summary, we identified 16,174 differentially expressed genes in longissimus muscle in 30M vs. Emb135 samples. According to the screening criteria chosen (FDR ≤0.001 and |log_2_Ratio| ≥1), we restricted these 16,174 genes to 6,800 genes that exhibited differential expression between the two tested samples. Of these, the expression levels of 1,893 genes were up-regulated in the Emb135 sample with respect to the 30M sample. The remaining 4,907 genes were down-regulated in the Emb135 sample with respect to the 30M sample (Figure 3 and Table S4).

### qRT-PCR Confirmation of Differential Gene Expression

To validate the expression profiles obtained using RNA-Seq, quantitative real-time PCR (qRT-PCR) was performed on 47 genes, which were randomly selected for high or low expression levels normalized to β-actin via qRT-PCR on pooled mRNA samples from Emb135 (n = 3), newborn (day 0 at birth, n = 3) and 30M (n = 3) cattle. Overall, of these 47 randomly selected genes, those that were differentially expressed showed similar patterns of mRNA abundance in the RNA-Seq analysis and qRT-PCR (Figure S1). Table S5 lists the primers used for the qRT-PCR of these 47 genes.

### Gene Ontology Analysis

**Figure 4:**
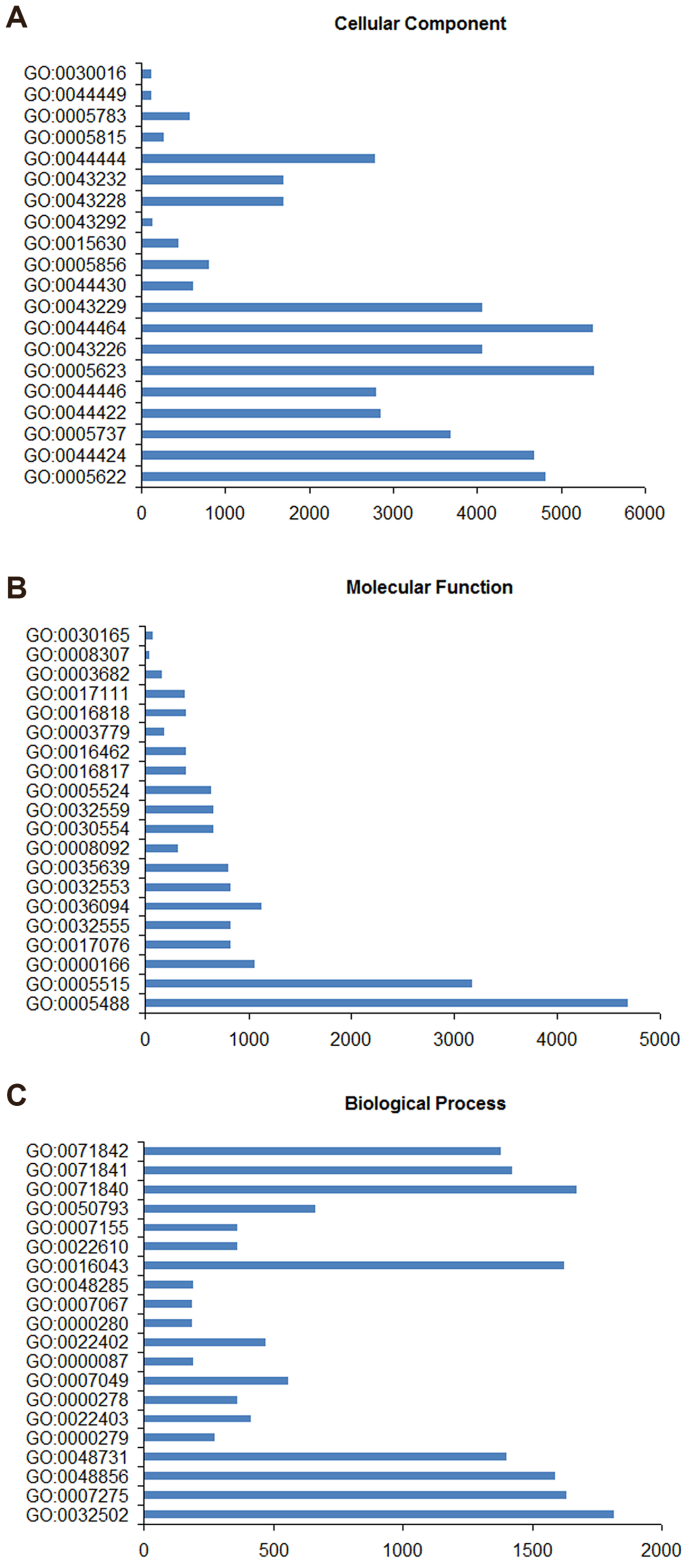
The top 20 Gene Ontology functional annotations for the differentially expressed genes. Bar graphs (A), (B) and (C) show three independent Gene Ontology (GO) information categories: cellular components, molecular functions and biological processes, respectively. In each bar graph, the abscissa represents the number of differentially expressed genes, and the ordinate is the ID number of the GO terms. All GO categories listed have enrichment *P* values <0.05.

To further investigate the biological processes associated with the differentially expressed genes, we performed Gene Ontology (GO) analysis by running queries for each differentially expressed gene against the GO database [20], which provides information on the relevant molecular functions, cellular components, and biological processes. The results of analyzing the GO functional annotations are presented in Figure 4 and Table S6. In total, 9,202 GO terms were assigned to the differentially expressed genes, and these were classified into three independent ontology categories: (1) 6,575 (71.5%) terms corresponding to biological processes, (2) 1,739 (18.9%) terms corresponding to molecular functions, and (3) 888 (9.7%) terms corresponding to cellular components. In the biological processes category, the most important enriched terms are related to developmental process, multicellular organismal development, and anatomical structure development; these were followed by system development, M-phase, cell cycle phase, mitotic cell cycle, and cell cycle. Binding and protein binding account for most of the terms in the molecular function category, followed by nucleotide binding, purine nucleotide binding, purine ribonucleotide binding, and small molecule binding. Finally, within the cellular component category, the GO term with the highest level of significance was intracellular. Furthermore, after intracellular, the terms intracellular part, cytoplasm, organelle part, intracellular organelle part, and cell exhibited the highest significance in the GO analysis.

### KEGG Pathway Analysis

Different genes usually cooperate with each other to exercise their biological functions. Pathway-based analysis helps to further understand the biological functions of genes. Pathway enrichment analysis identifies significantly enriched metabolic pathways or signal transduction pathways related to differentially expressed genes. Overall, 254 pathways were assigned for differentially expressed genes. 15 pathways were significantly enriched (Qvalue ≤0.05) for differentially expressed genes (Table S7). The pathway term showing the highest level of significance was axon guidance. Furthermore, after axon guidance, the terms phagosome, cell cycle, hypertrophic cardiomyopathy (HCM), dilated cardiomyopathy, pathways in cancer, amyotrophic lateral sclerosis (ALS), DNA replication, N-glycan biosynthesis, and regulation of actin cytoskeleton exhibited the highest significance in the pathway analysis.

### Detecting SNP Variants in Pooled Transcriptome Samples by RNA-Seq

**Figure 5:**
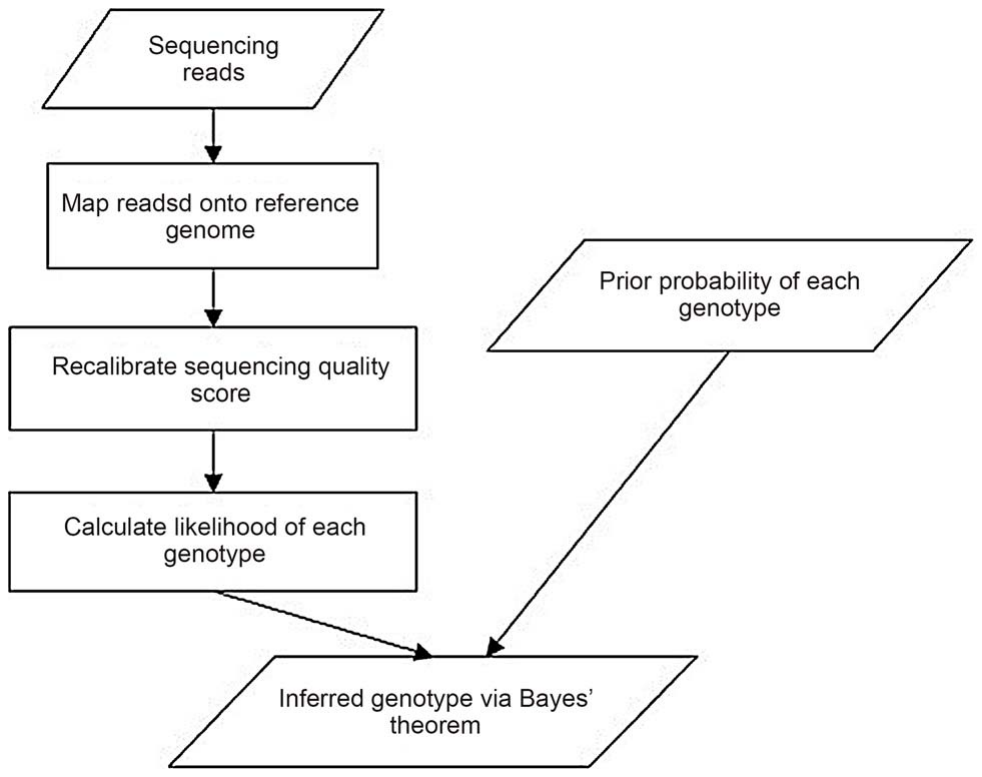
Pipeline of SNP Calling. The SNPs can be identified on the consensus sequence through the comparison with the reference.

The SOAPsnp program [21] calculates the likelihood of each genotype at each site based on the alignment of short reads to the UMD 3.1 reference genome together with the corresponding sequencing quality scores. The program then infers the genotype with highest posterior probability at each site based on Bayes' theorem (the reverse probability model). Thus, we have accounted for the intrinsic bias or errors that are common in Illumina sequencing data and recalibrated the quality values for use in inferring consensus sequences. Figure 5 summarizes the workflow used for the discovery of putative SNPs. According to this workflow, 31,334 putative SNPs were identified for the 30M pooled samples. Similarly, for Emb135 pooled samples, 30,618 putative SNPs were identified. For both samples, the SNP detection stringency conditions were as follows: (1) at least two unique mapping reads that support the polymorphic nucleotide and (2) a quality score of ≥20 (Table S8).

## Discussion

The unprecedented level of sensitivity and high throughput of deep sequencing technologies suggest that RNA-Seq is likely to become the platform of choice for transcriptome analysis, quickly superseding microarray-based methods for comprehensive studies of novel transcribed units and differential splicing activity, and for the refinement of gene structures, the measurement of gene expression levels and the discovery of expressed SNPs. However, the new level of detail offered by RNA-Seq raises novel statistical and computational challenges that limit the widespread adoption of whole-genome transcriptional profiling to detect genes that are differentially expressed among several experiments.

In this study, we generated >2 billion sequence reads, corresponding to 2.7 Gb of raw sequence data, using Illumina sequencing of mRNA from two stages of longissimus muscle development. Aligning all sequence reads to the bovine UMD 3.1 reference genome yielded >20 million reads that were successfully mapped on the UMD 3.1 reference genome. More than 5 million reads remain that cannot be matched to the UMD 3.1 reference genome. This could be caused by low sequence coverage of the reference genome, reference errors, sequencing errors, or defined mapping criteria. A majority of the annotated transcripts in the Ensembl database were covered by sequenced reads, illustrating the sensitivity of RNA-seq for transcript discovery, even for genes expressed at low levels [22]. Furthermore, we identified 24,464–29,994 novel transcript units, which improved the gene annotations of the bovine genome and transcriptome.

Alternative splicing is an important model of gene expression regulation that has not been generally accessible through microarray or SAGE analysis methods in cattle [23]. We found that some genes showed all four types of alternative splicing models, thus revealing the complexity of alternative splicing in cattle. We also found that alternative 3′-end splicing is the most common type of alternative splicing event in cattle and that intron retention is the least common alternative splicing mechanism. Our results are consistent with those of a similar pig study [24]. However, these results contrast with those reported for humans and Holstein cows, where exon-skipping is the most prevalent mechanism [25], [26]; the results also differ from those found for rice, for which intron retention is the primary alternative splicing mode [27]. Three types of alternative splicing, including alternative first exon-, alternative last exon- and mutually exclusive exon-splicing, were excluded from our analyses because the algorithms used to analyze these have not yet been perfected. A greater number of alternative spliced genes would be discovered if these alternative splicing models were considered.

With the rapid rise of high throughput sequencing, a plethora of transcriptomic data is now available, in particular for mammals. Comparing the transcriptomes of beef cattle (Qinchuan beef cattle were analyzed in this study) and dairy cattle (Holstein cows were analyzed in a similar study [26]), we observed that the distribution of genes based on the GO terms within each ontological category derived for beef cattle is consistent between these two breeds. Indeed, in both breed types, the most represented terms in the biological process category are developmental process, multicellular organismal development, anatomical structure development, and system development. Although a few differences exist within each category, the general organization and the main terms found are similar between both breeds. However, metabolic process, cellular metabolic process, catalytic activity, and oxidoreductase activity are the major terms found in the biological process category for porcines. In addition, cellular process, metabolic process and biological regulation, multicellular organismal process, and developmental process are the main terms representing the biological process ontological category in amphioxus. These results indicate that similar species are likely to have a similar general organization for each ontological category of genes. Conversely, distantly related species are likely to have considerable differences in the general organization for each ontological gene category.

In conclusion, we sequenced the transcriptome of Qinchuan beef cattle using the RNA-seq method. This study revealed a large number of genes of known and unknown function, greatly expanding the amount of genetic information available for this species and providing a profile of its developmental processes. This study also represents a first step toward an improved understanding of the functions of these genes and provides a broad and novel vision of future research at the molecular level in cattle.

## Materials and Methods

### Ethics Statement

The Qinchuan beef cattle used in this experiment were obtained from the Shaanxi Kingbull animal husbandry company limited, Baoji City, China. Experiments were performed according to the Regulations of the Administration of Affairs Concerning Experimental Animals (Ministry of Science and Technology, China, revised in 2004) and approved by the Institutional Animal Care and Use Committee of the College of Animal Science and Technology, Northwest A&F University, China. Animals were allowed access to feed and water *ad libitum* under same, normal conditions and were humanely killed.

### RNA Isolation

Liver, lung, spleen, kidney, heart, stomach, intestines, fat (only newborn and 30M) and longissimus muscle from 9 individual were harvested for RNA isolation within 10 minutes after slaughter (3 individuals at each of three key stages of myogenesis and muscle maturation: Emb135d, newborn, and 30M adult cattle. All fresh tissue samples were collected and divided into 1.5 mL plastic centrifuge tubes (each sample weighing around 100 mg) and snap frozen in liquid nitrogen for RNA-seq (longissimus muscle samples for RNA-seq were processed only for Emb135d and 30M cattle) and qRT-PCR use.

Total RNA was isolated using TRIzol reagent (Invitrogen, USA) according to the manufacturer's instructions. Briefly, 1 mL of TRIzol was added to the pellet after removal of the 1.5 mL plastic centrifuge tube from the liquid nitrogen and immediately homogenized by pipetting; thereafter, RNA were isolated and purified at 4°C using standard phenol–chloroform extraction. DNA was removed from the RNA extracts by incubation with RNase-free DNase I (Qiagen, Hilden, Germany) for 1 h at 37°C. RNA concentration and purity were determined using a NanoDrop ND-1000 spectrophotometer (Nanodrop, Wilmington, DE), and its quality and integrity were assessed based on the A260/A280 ratio and confirmed using a 2100 Bioanalyzer (Agilent) according to RNA integrity number. Equal amounts of high-quality RNA samples from each tissue were then pooled for cDNA synthesis and sequencing. Specifically, longissimus muscle from individuals at embryo (n = 3) and adult (n = 3) stages were pooled as samples Emb135 and 30M, respectively, for use in sequencing.

### cDNA Preparation and Library Construction for Illumina Sequencing

Messenger RNA was isolated from the total RNA samples using oligo (dT) magnetic beads (Invitrogen). The purified mRNA was first fragmented to approximately 200–700 nt fragments using the RNA fragmentation kit (Ambion). First-strand cDNA synthesis was performed using random hexamer primers and reverse transcriptase. After the first strand was synthesized, a custom second-strand primer and strand synthesis buffer (Illumina) were added, followed by dNTPs, RNase H and DNA polymerase I to nick translate the second-strand. The reaction was then purified using a QiaQuick PCR column (Qiagen) and eluted in EB buffer (Qiagen); the samples were then subjected to end repair, addition of a single A base, adaptor ligation, agarose gel isolation of approximately 200-bp cDNA, and PCR amplification. Finally, the samples were sequenced using the Illumina HiSeq™ 2000 platform. Overall, two cDNA libraries were constructed from pooled Emb135 (n = 3) and 30M (n = 3) samples. One technical replicate was performed for each sample.

### Read Mapping on the Bovine Reference Genome and Data Analysis

Illumina sequencing of the Emb135 and 30M pooled RNA was conducted in BGI, Shenzhen, China. The UMD 3.1 reference genome (available at ftp://ftp.ensembl.org/pub/release-67/fasta/bos_taurus/) from the Ensembl database was utilized as the bovine reference genome for the assembly [28]. After trimming reads containing adaptors, reads containing more than five Ns per 100 bp and reads of low quality (i.e., more than half of the base's qualities were less than or equal to 5), clean reads were 100% matched to the UMD 3.1 reference genome using SOAP2 [13] by not allowing any mismatches in reads. For the reads that were not able to be aligned with the reference sequences, we performed the SOAP alignment again while allowing less than 3 bp mismatches in the reads.

A similar strategy was used to filter the unique sequence reads and multi-position reads. Data were normalized by calculating the number of reads per kilobase of exon region in a gene per million mapped reads (RPKM) (RPKM  =  total exon reads/mapped reads in millions × exon length in kb) [4] for each gene and then annotated with the Ensembl bovine genome assembly.

### Identification of Novel Transcript Units

All reads that matched the UMD 3.1 reference genome with multi-positions were excluded from further analysis. The intergenic regions were defined as regions between 200 bp downstream of one gene and 200 bp upstream of the next adjacent gene using the bovine mRNA data (Ensembl). A contiguous expression region with each base supported by at least two reads was considered a transcriptionally active region. Transcriptionally active regions that were joined by at least one set of paired-end reads were connected into a single transcript unit. Transcript units that were not overlapped with an annotated gene model and located in intergenic regions with a continuous mapping length ≥150 bp and average coverage ≥2 were considered as putative novel transcript units.

### Identification of Alternative Splicing Models

As described by Wang et al. [29] and Zhang et al. [27] and according to the structure of exons, alternative splicing events were classified into seven different types of alternative splicing models: exon skipping, intron retention, alternative 5′ splice site, alternative 3′ splice site, alternative first exon, alternative last exon, and mutually exclusive exon models. The details of these alternative splicing models have been described by Zhang et al. [27]. Because the algorithms for the alternative first exon, alternative last exon and mutually exclusive exon models have not yet been perfected, only the remaining four alternative splicing models listed above were analyzed and presented in this study.

### Refinement of Gene Structures

Gene structure was optimized according to the distribution of reads, paired-end sequences and reference gene annotations. After aligning reads to the UMD 3.1 reference genome, the genomic regions with continuous reads and uniquely mapped reads ≥2 formed transcription-active regions. We connected the various transcriptionally active regions to form a potential gene model using the paired-end data. The extensions of the 5′ and 3′ boundaries were determined by comparing the potential gene model with the existing gene annotation.

### Analysis of Differentially Expressed Genes

Gene expression level was normalized by considering RPKM value [4]. Differentially expressed genes and their corresponding *P*-values were determined using the methods described by Audic and Claverie [30]. The significance threshold of the *P*-value in multiple tests was set based on the FDR. The fold changes (log_2_Ratio) were also estimated according to the normalized gene expression level in each sample. We used “FDR ≤0.001 and |log_2_Ratio| >2” as the threshold to judge the significance of gene expression differences.

### Gene Ontology Annotation

The differentially expressed genes were classified into the categories of cellular component, molecular function, and biological process using the GO annotation. The hypergeometric test was applied to map all differentially expressed genes to terms in the GO database (http://www.geneontology.org/) [20] and to search for significantly enriched GO terms in differentially expressed genes by comparing them to the genome background. The calculated *P*-values were corrected using the Bonferroni correction, taking the corrected *P*-value ≤0.05 as the significance threshold.

### KEGG Pathway

KEGG is the major public pathway-related database. Pathway-based analysis helps to further understand the biological functions of genes. The calculation formula is the same as that used for the GO analysis. Pathway enrichment analysis identifies significantly enriched metabolic pathways or signal transduction pathways in differentially expressed genes by comparison against the whole genome background. The calculated *P*-values were corrected using the Bonferroni correction, taking pathways with Qvalues ≤0.05 as significantly enriched in differentially expressed genes.

### Detection of Putative SNPs

During our experiment, we used SOAPsnp software [21] to detect SNPs between two samples based on the massively parallel Illumina GA technology. SOAPsnp is a member of the SOAP (Short Oligonucleotide Analysis Package) software. The program is a resequencing utility that can assemble consensus sequences for the genome of a newly sequenced individual based on the alignment of the raw sequencing reads to a known reference genome. The SNPs can then be identified in the consensus sequences by a comparison with the reference.

### Validation of RNA-Seq Data

We used qRT-PCR, the current gold standard for the quantification of mRNA, to validate the repeatability and reproducibility of gene expression data obtained by RNA sequencing in cattle. 47 differentially expressed genes with the highest significance (as identified by RNA-seq) were chosen for validation (the set includes both up- and down-regulated genes). As described in the methods section dealing with RNA isolation, expression was measured in eight (or nine) different tissue samples from three pooled individual embryos, newborns, and adults, respectively. Measurements were averaged over all tissues and compared to RNA-Seq results. The first-strand cDNA was synthesized using superscript II reverse transcriptase (Takara). Gene-specific primers were designed based on the gene sequence using Primer Premier 5.0 (Premier Biosoft) software. All primers for validation were designed to cross exon-exon junctions and are shown in Table S5. The cDNA was diluted 1∶10 in RNA-free water for target gene measurements, with the β-actin gene used as a control. qRT-PCR reactions were performed in triplicate using SYBR Premix Ex Taq^TM^ II (Takara) on an Bio-Rad CFX 96 Real-Time PCR Detection system (Bio-Rad Laboratories, Foster City, USA) using the following program: 95°C for 30 sec, then 40 cycles at 95°C for 5 sec, 60°C for 30 sec, 72°C for 45 sec, followed by a final extension step at 72°C for 5 min. The relative gene expression values were calculated using the 2^−ΔΔCt^ method [31].

## Supporting Information

Figure S1 **Comparison of changes in 47 differentially expressed genes from qRT-PCR in three developmental stages.**
(DOC)

Table S1 **Novel transcript units identified in Emb135 and 30M pooled samples.**
(XLS)

Table S2 **The number of each type of alternative splicing events in longissimus muscle tissues.**
(XLS)

Table S3 **The detailed description of the 5′ and 3′ end extension of gene in each sample.**
(XLS)

Table S4 **Differentially expressed genes in Emb135-VS-30M.**
(XLS)

Table S5 **Primers for Real-time RT-PCR of 47 DEG genes randomly selected for high or low expression levels.**
(XLS)

Table S6 **Gene Ontology (GO) assignment of the bovine transcriptome for the biological process, molecular function and cellular component category.**
(XLS)

Table S7 **List of KEGG pathway categories.**
(XLS)

Table S8 **Detection of putative SNPs.**
(XLS)
