# Supplementary material for: Characterization of Transcriptional Complexity during Longissimus Muscle Development in Bovines Using High-Throughput Sequencing
Source: PLoS One. 2013 Jun 7;8(6):e64356. doi: 10.1371/journal.pone.0064356 (PMC3676445; doi:10.1371/journal.pone.0064356)
Supplement: Figure S1 — Comparison of changes in 47 differentially expressed genes from qRT-PCR in three developmental stages. (DOC) [file pone.0064356.s001.doc]

**Figure S1. Comparison of changes in 47 differentially expressed genes from qRT-PCR in three developmental stages.** White bar shows fetal bovine; Blue bar represents newborn bovine; Red bar indicates adult bovine. Error bar represents standard error of three different biological replicates.


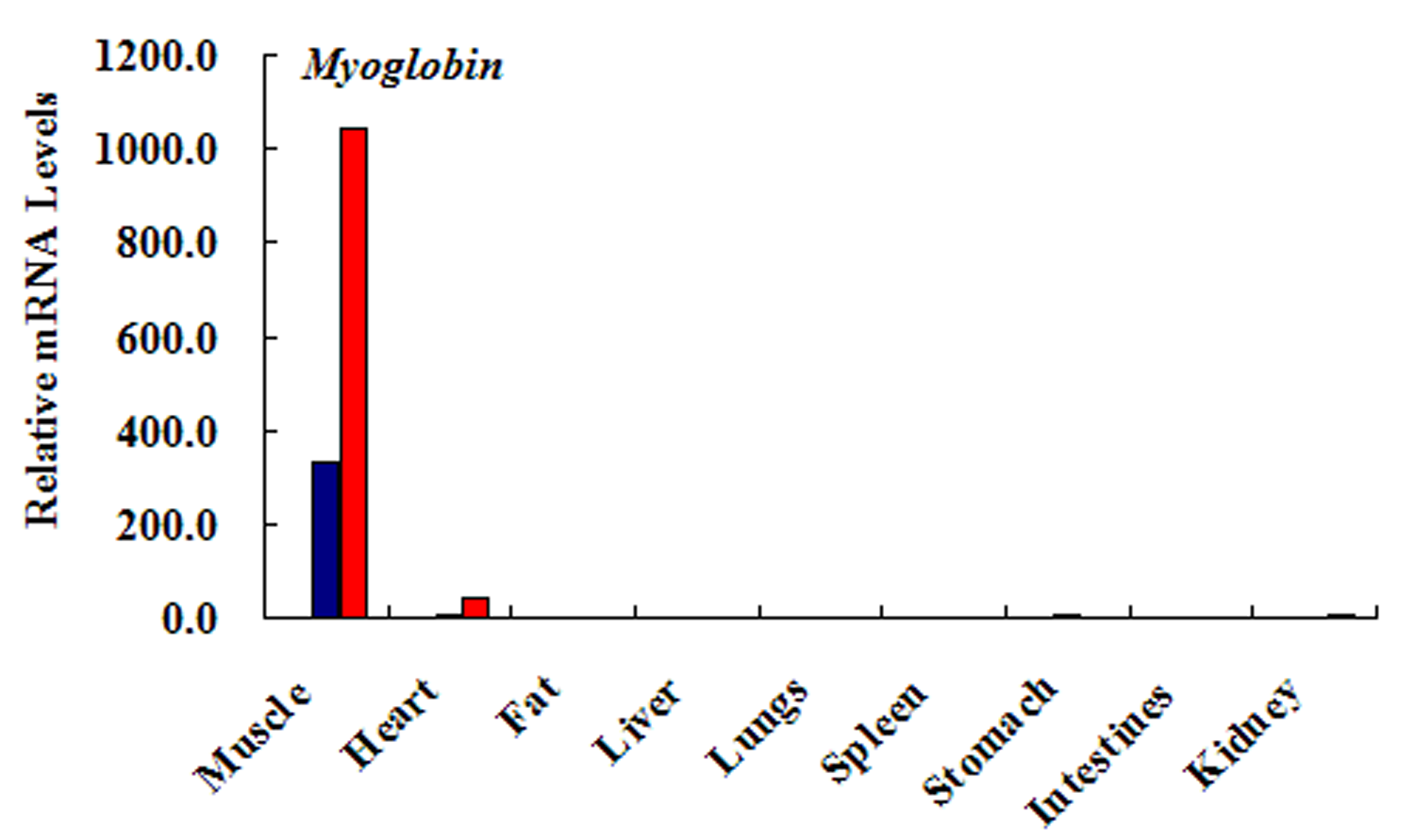

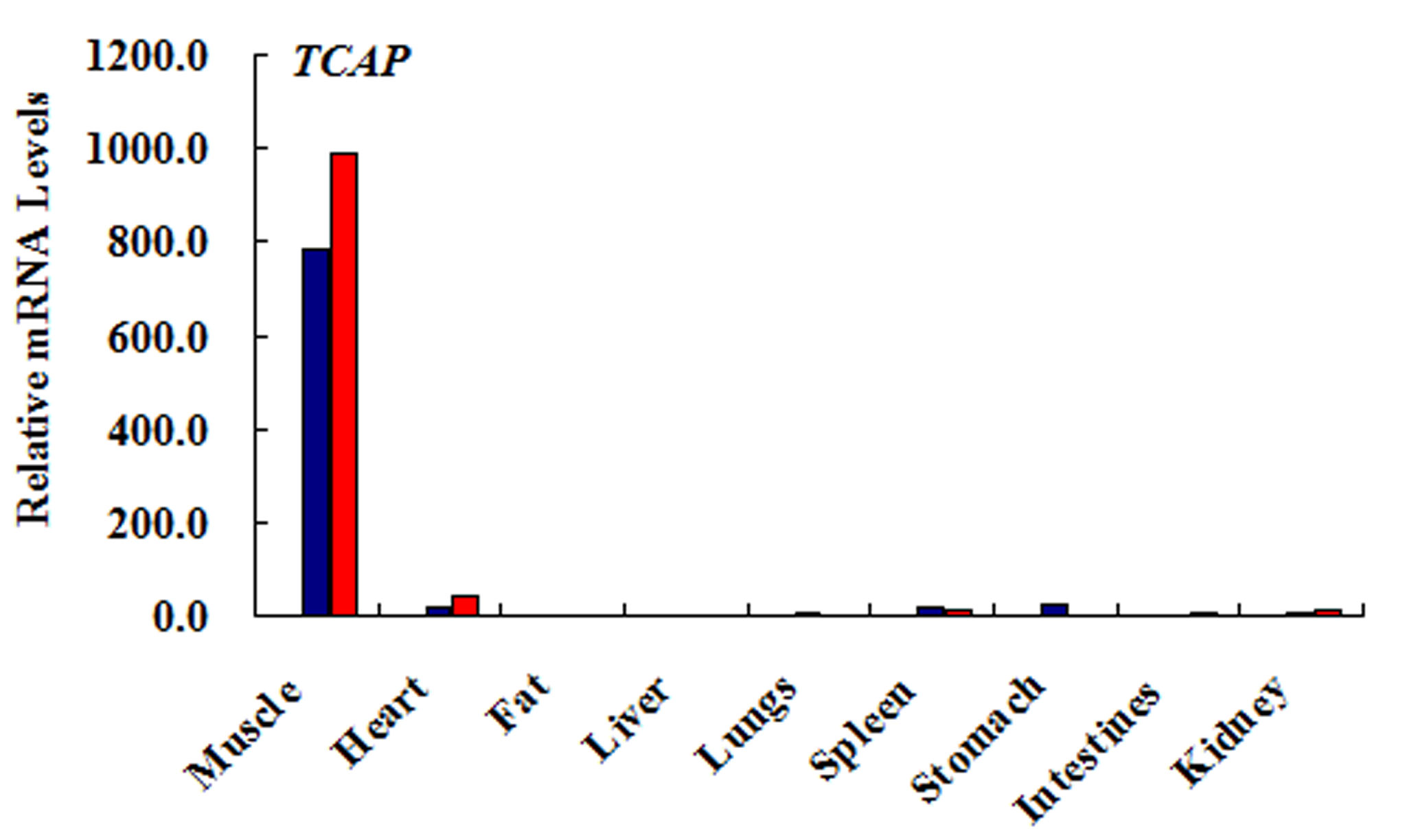


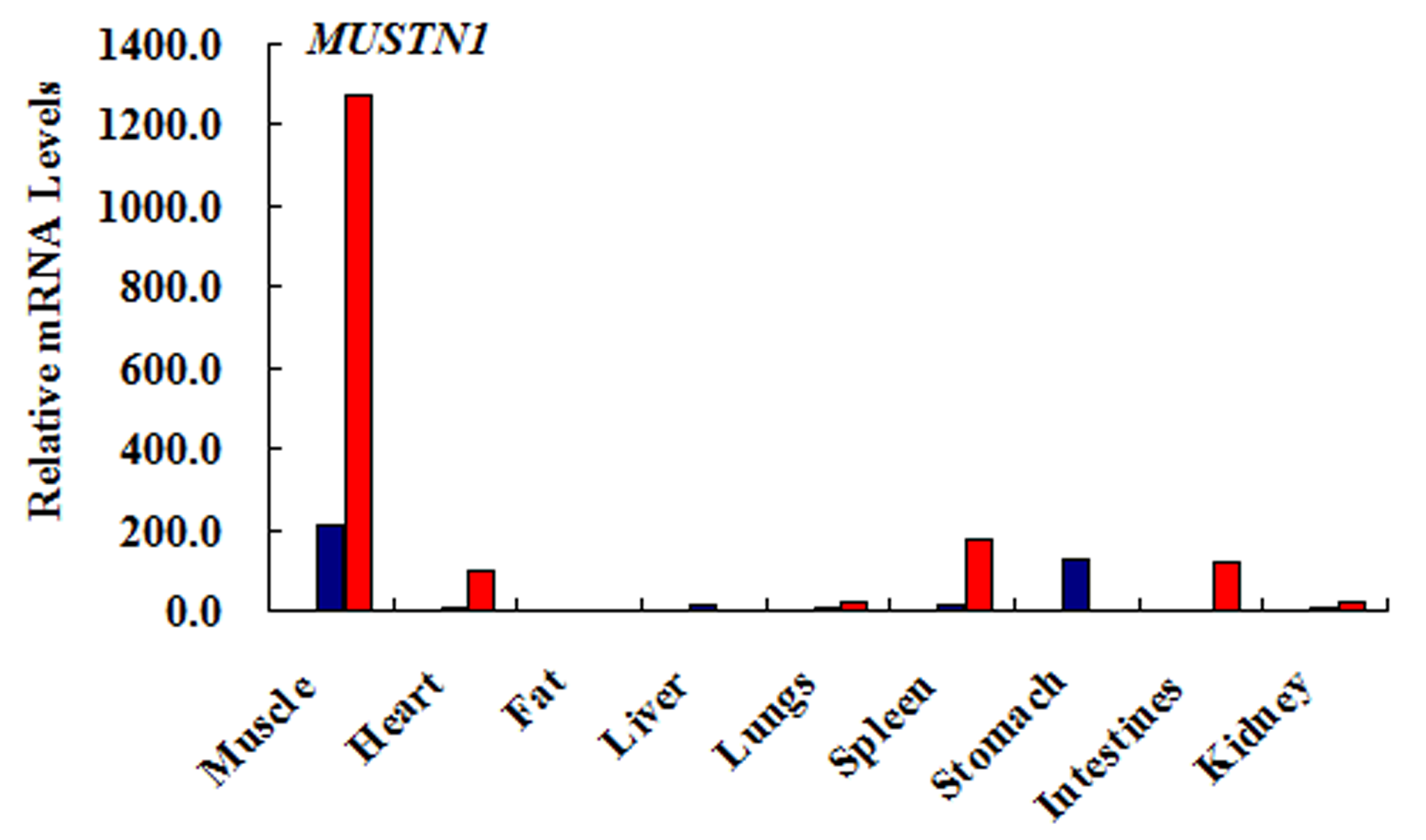

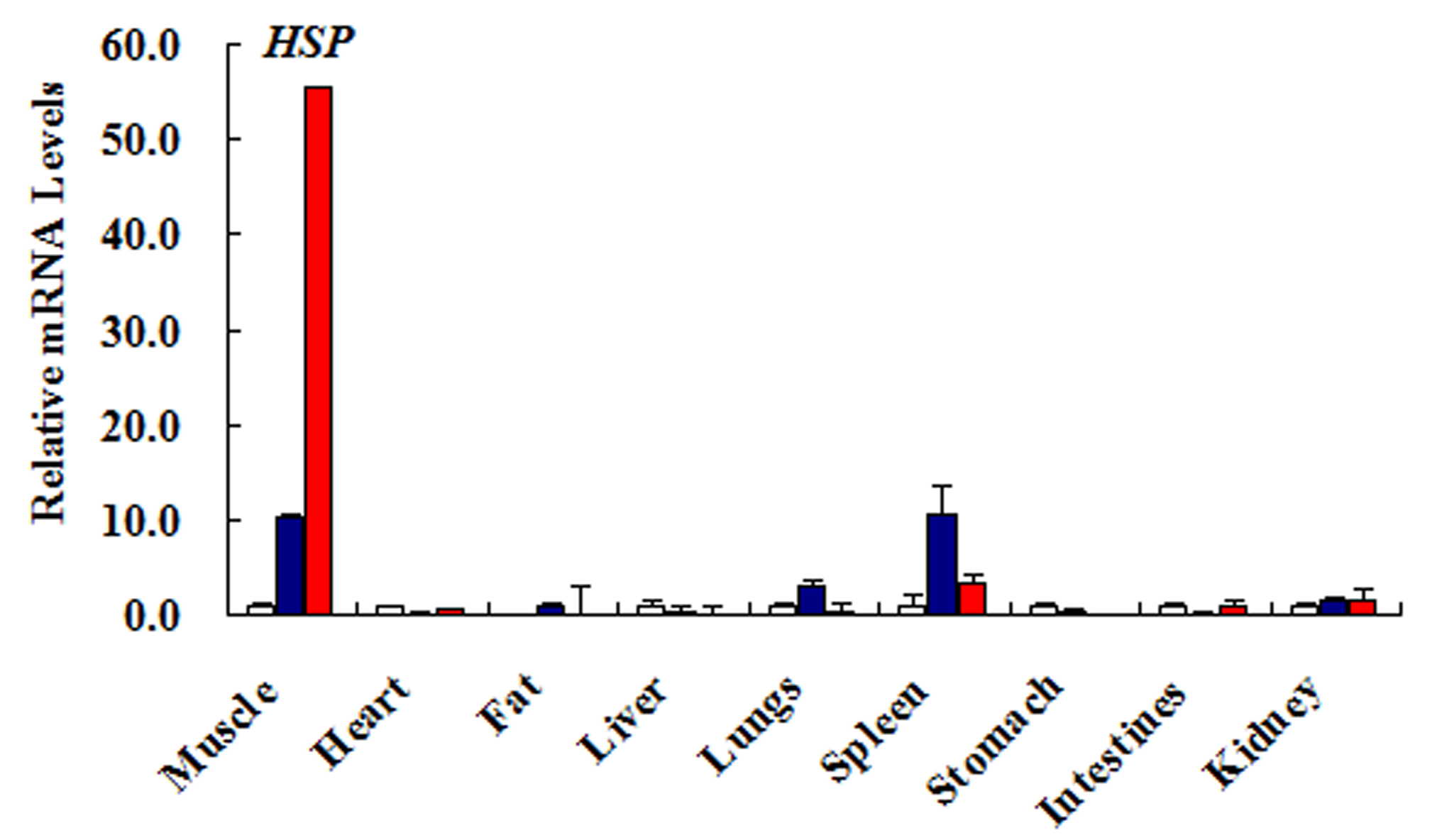


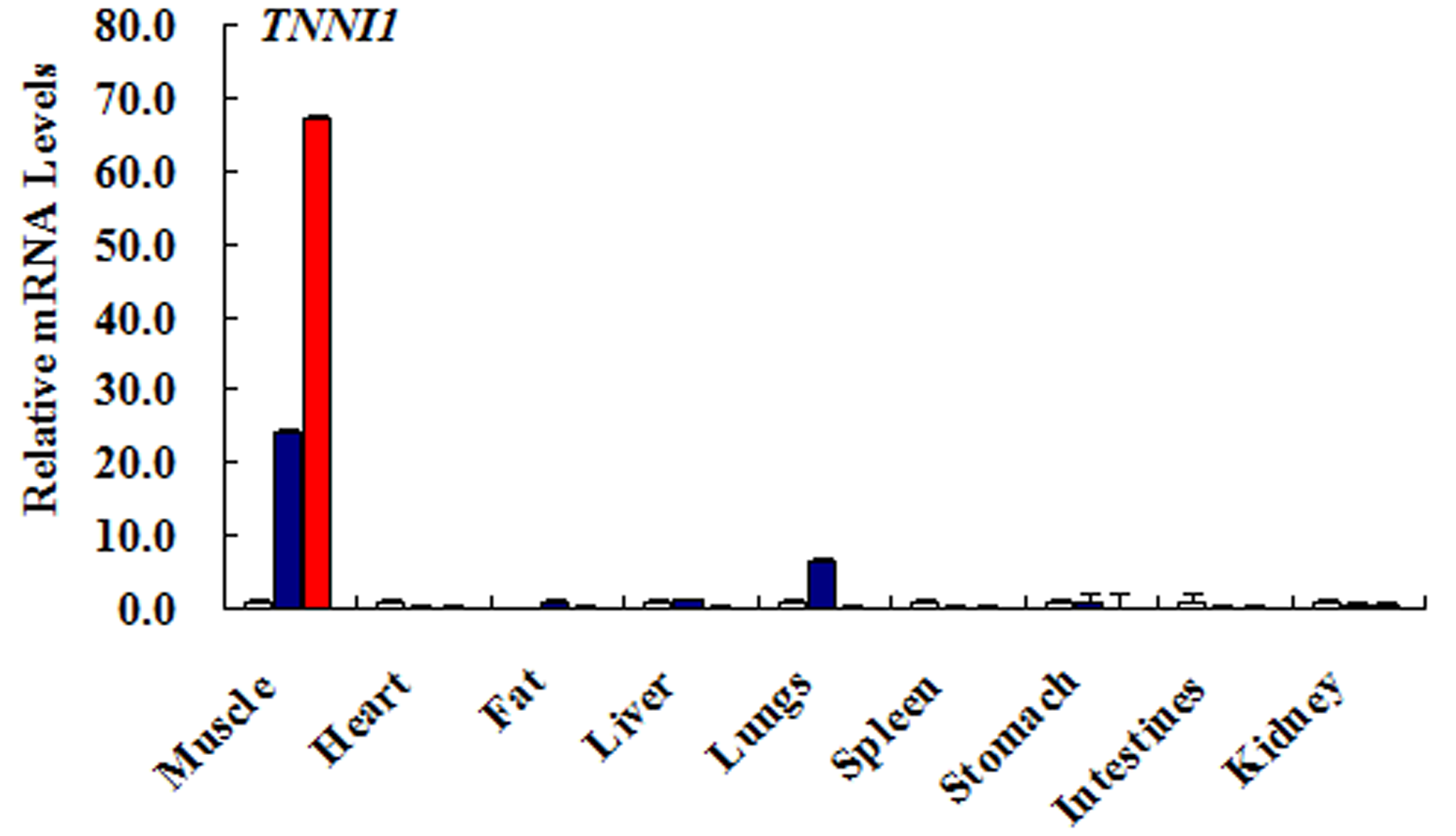

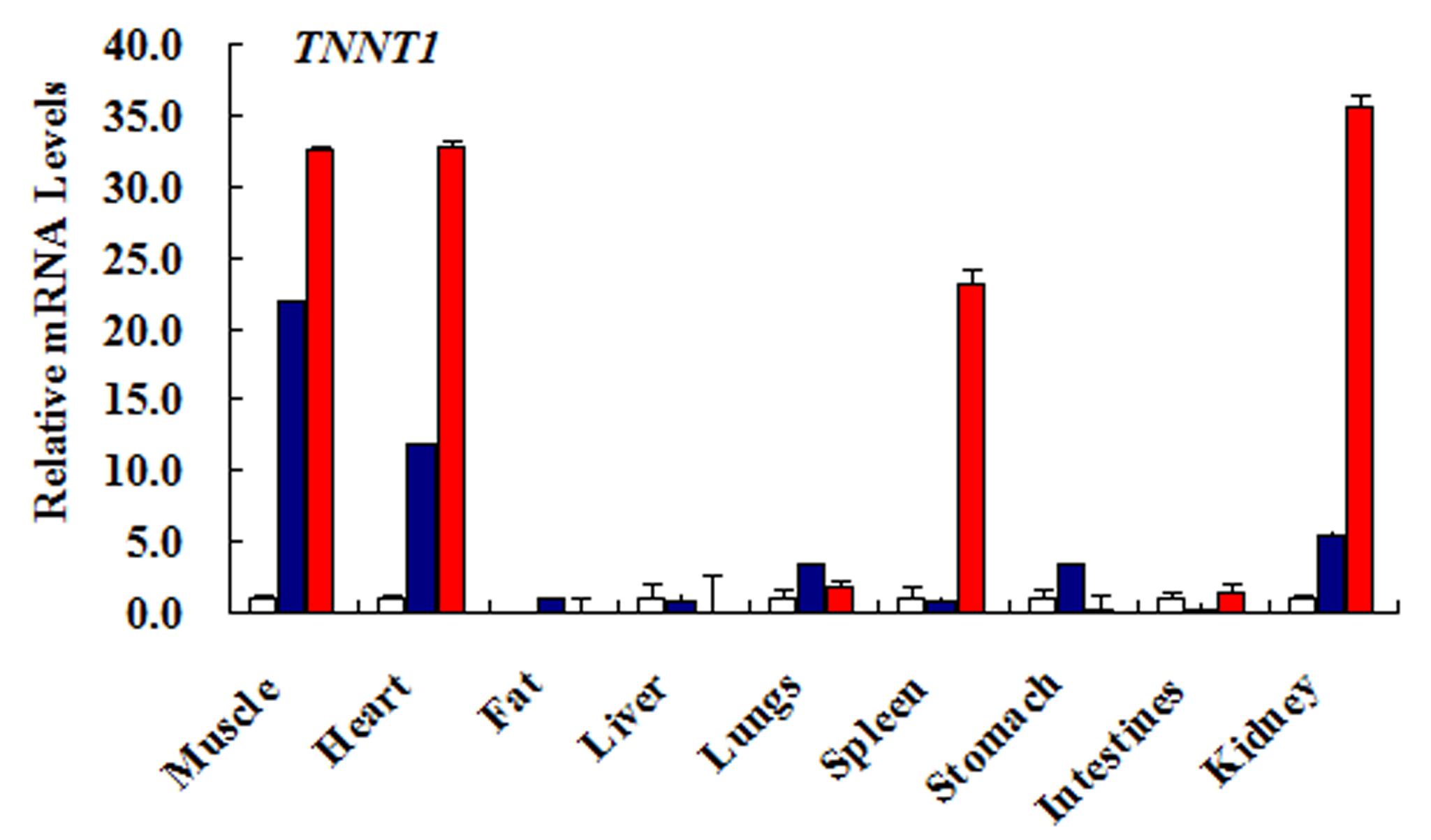


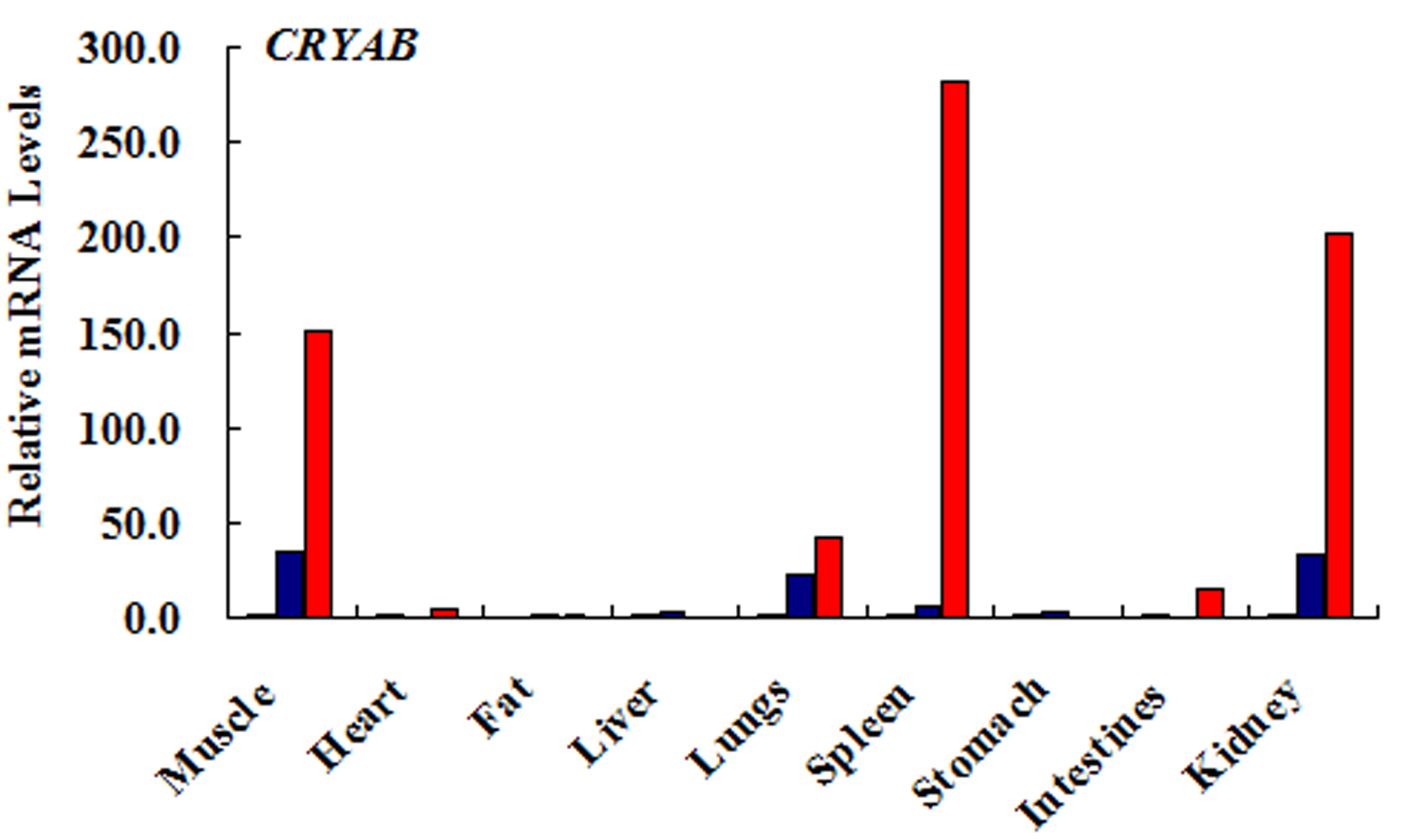

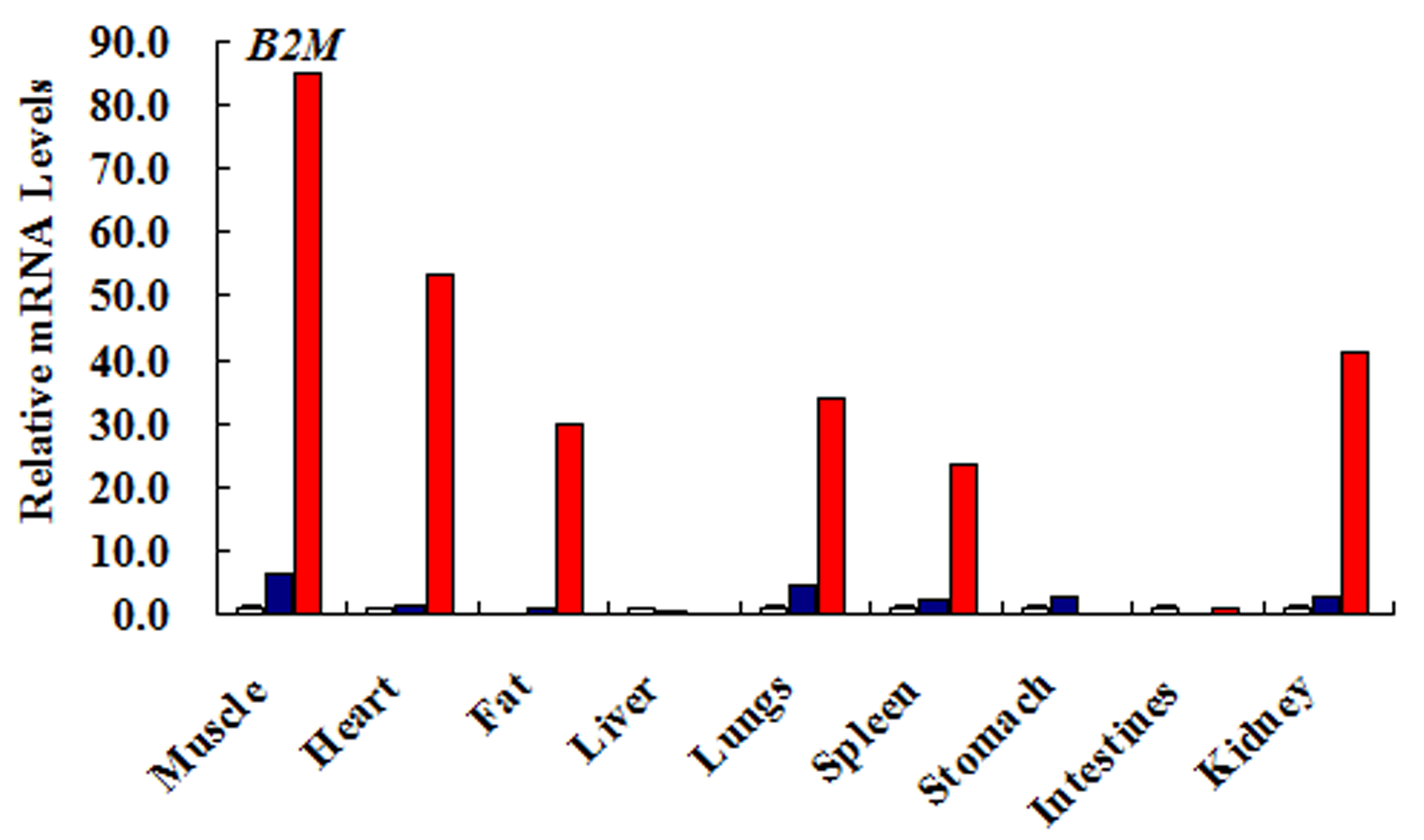


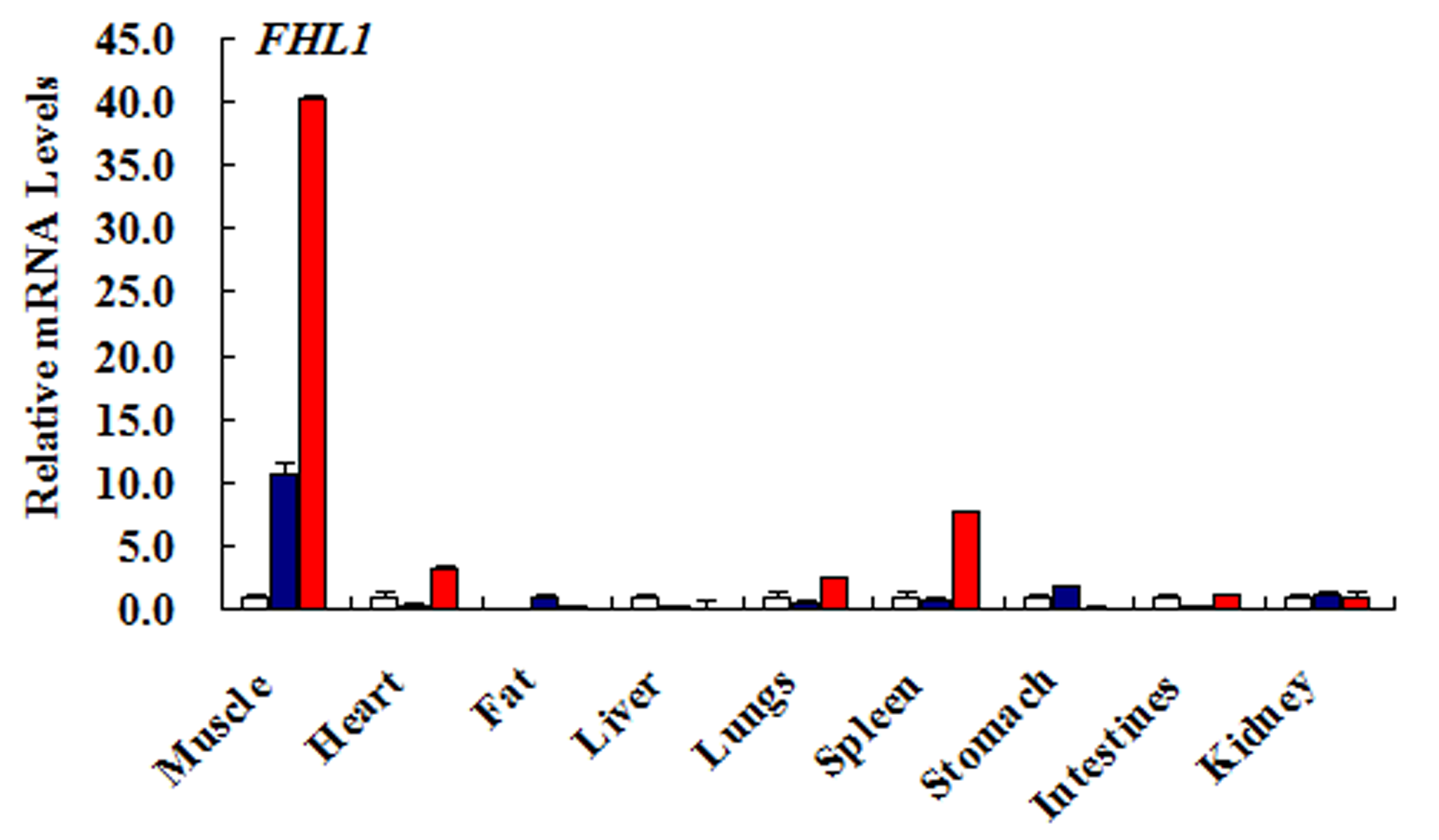

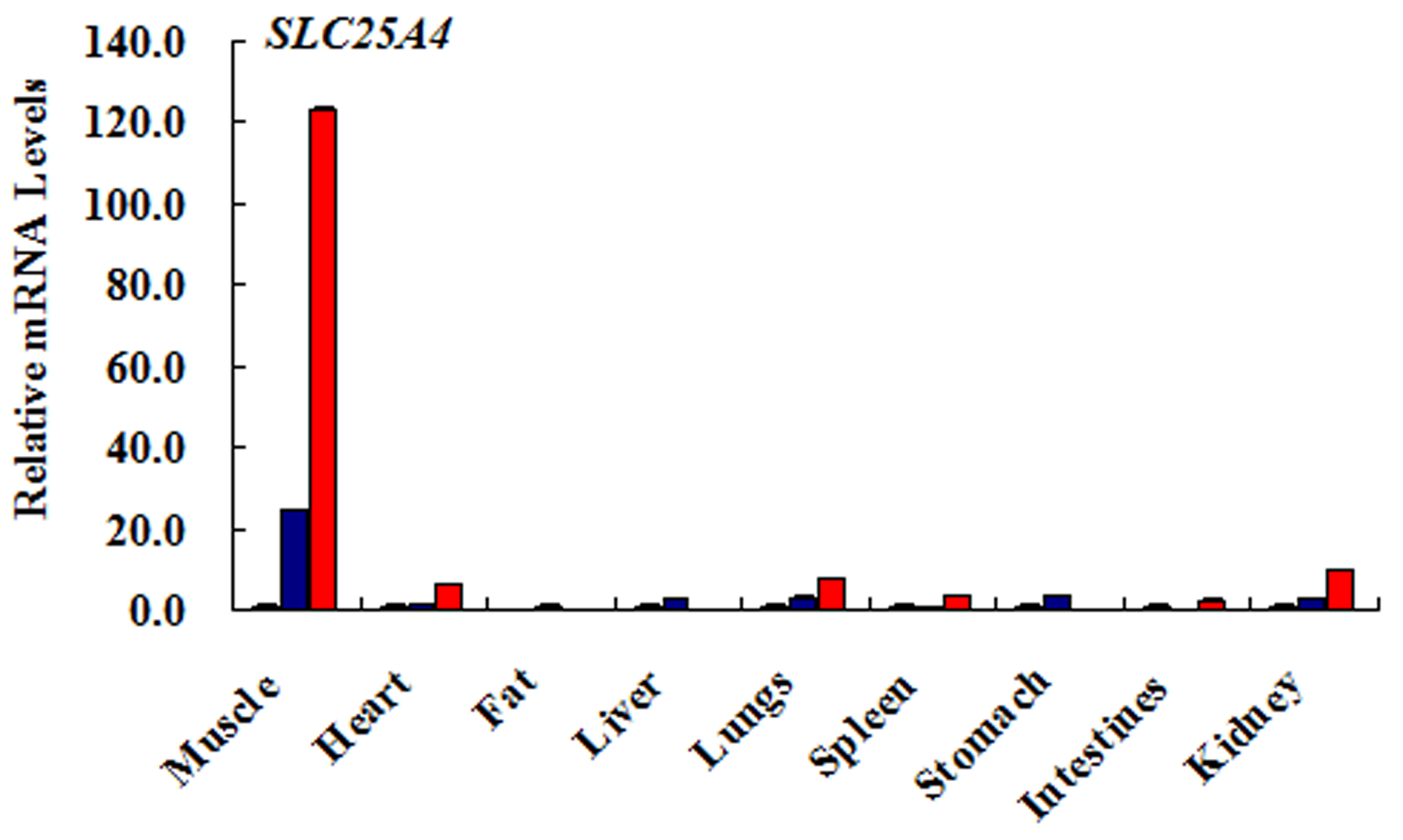


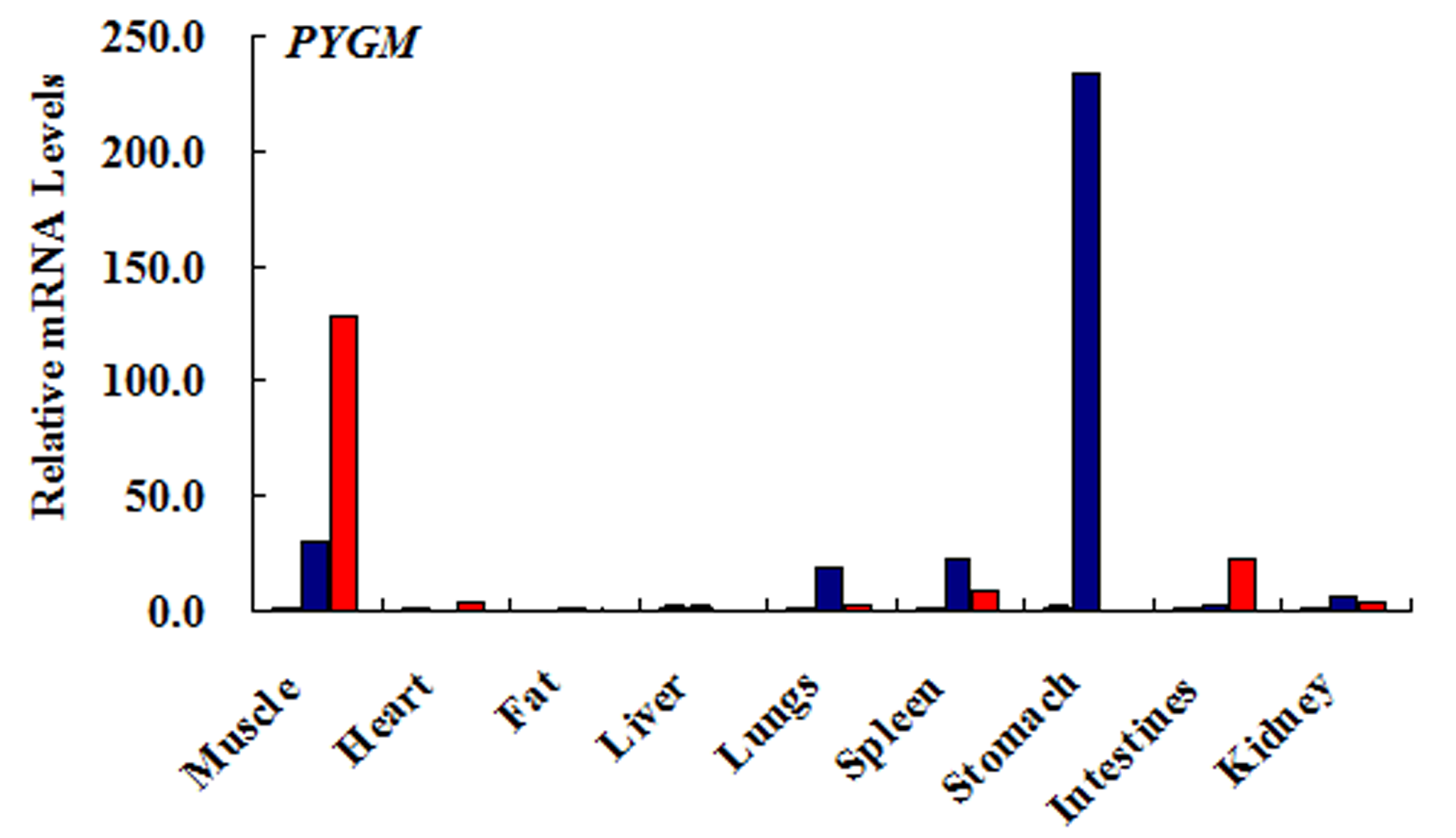

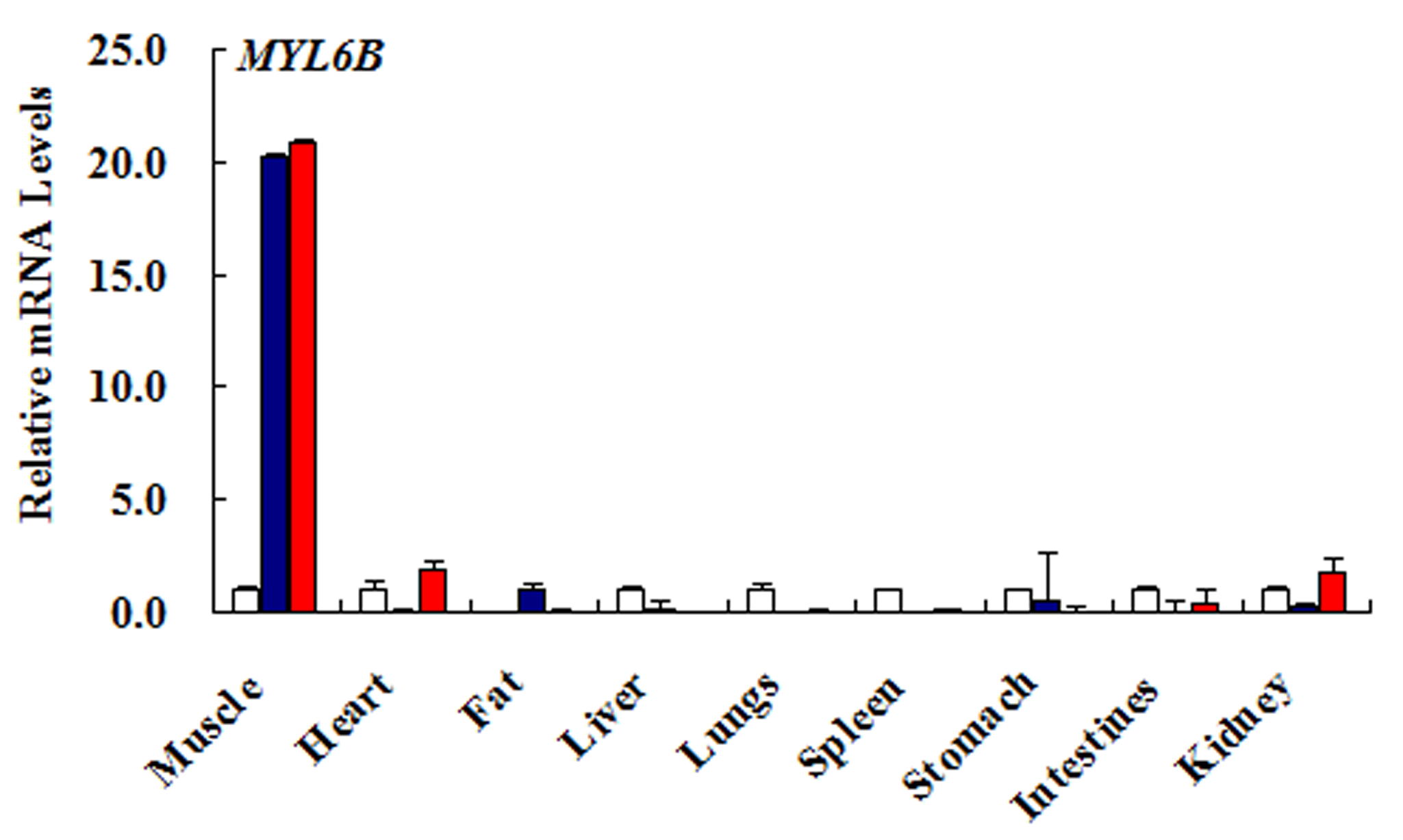


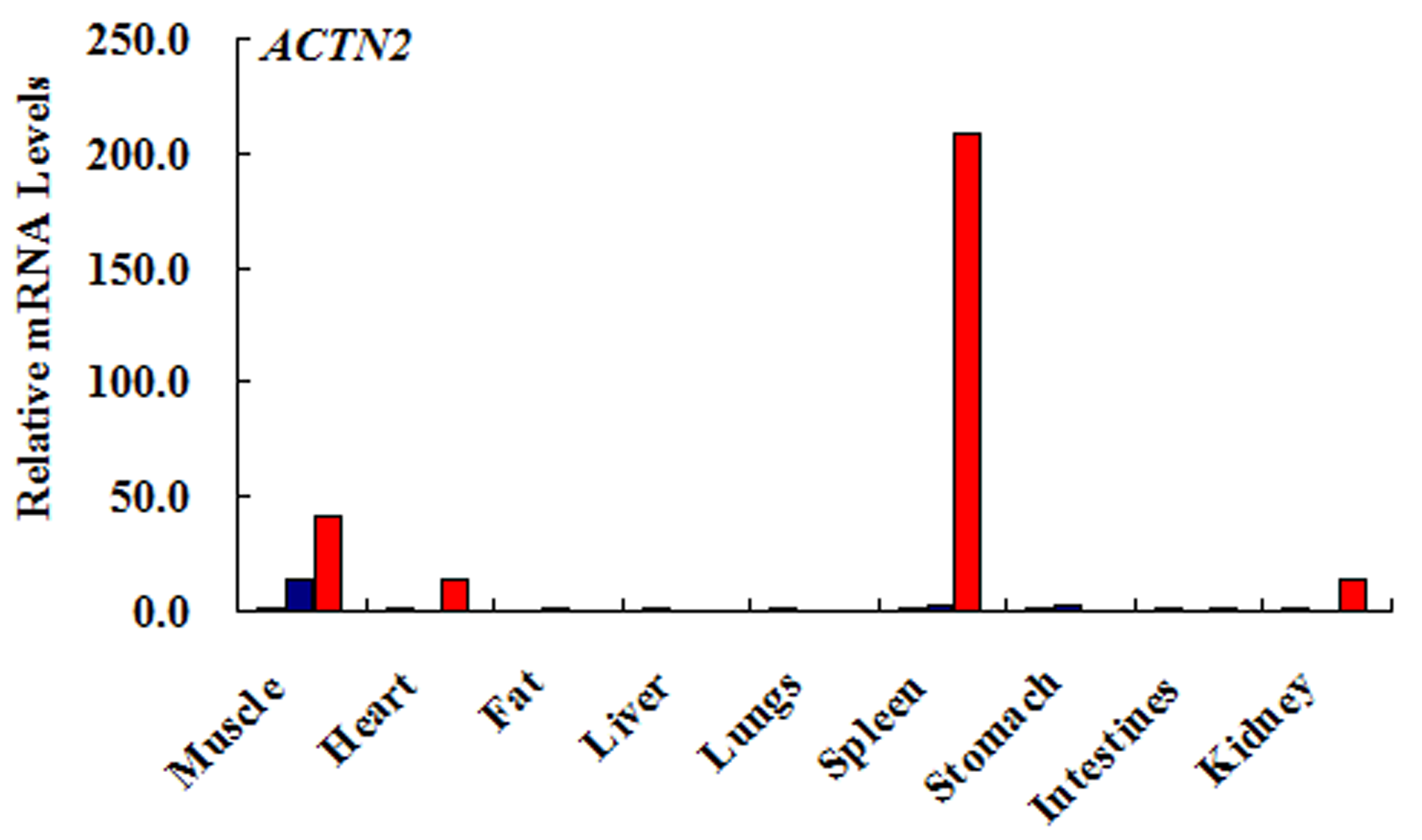

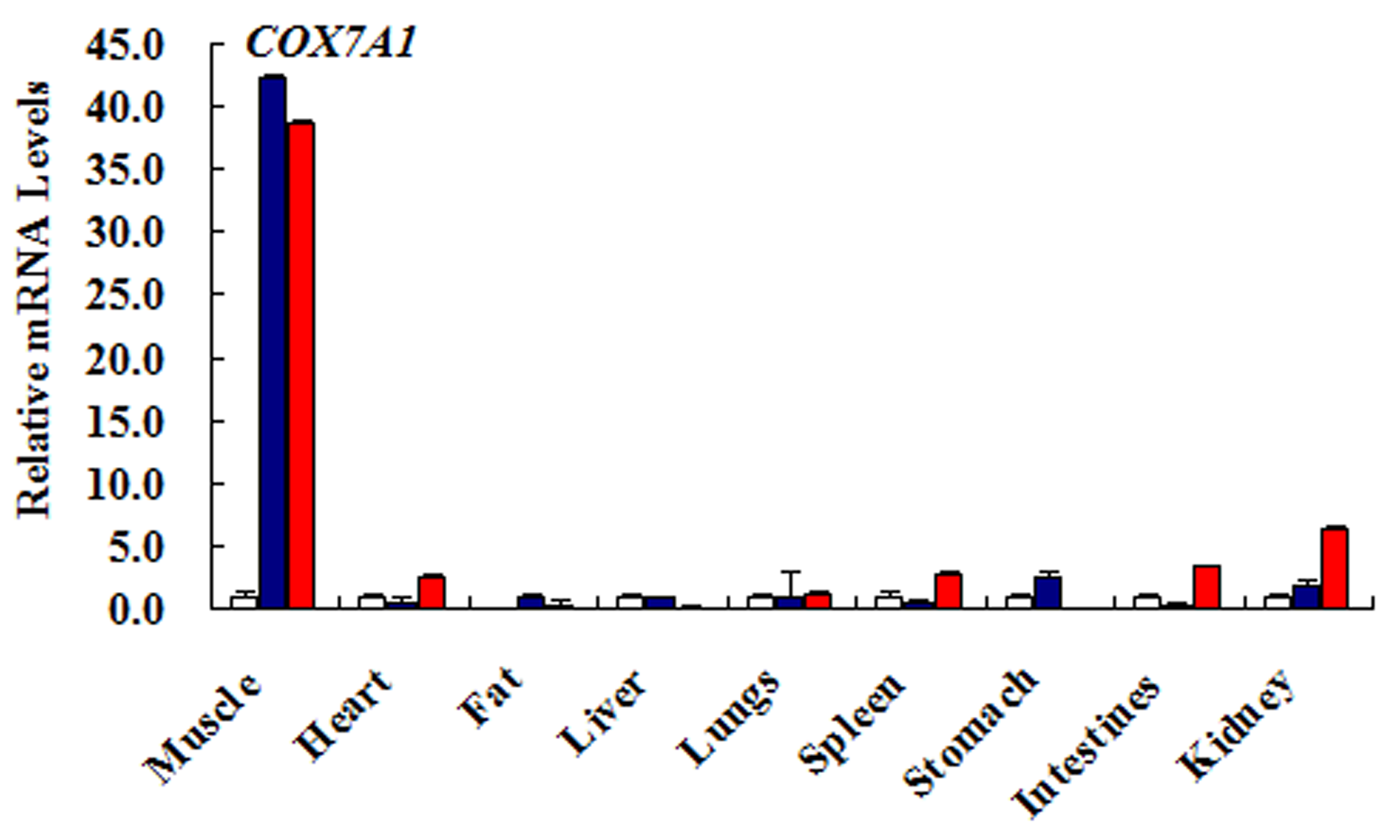


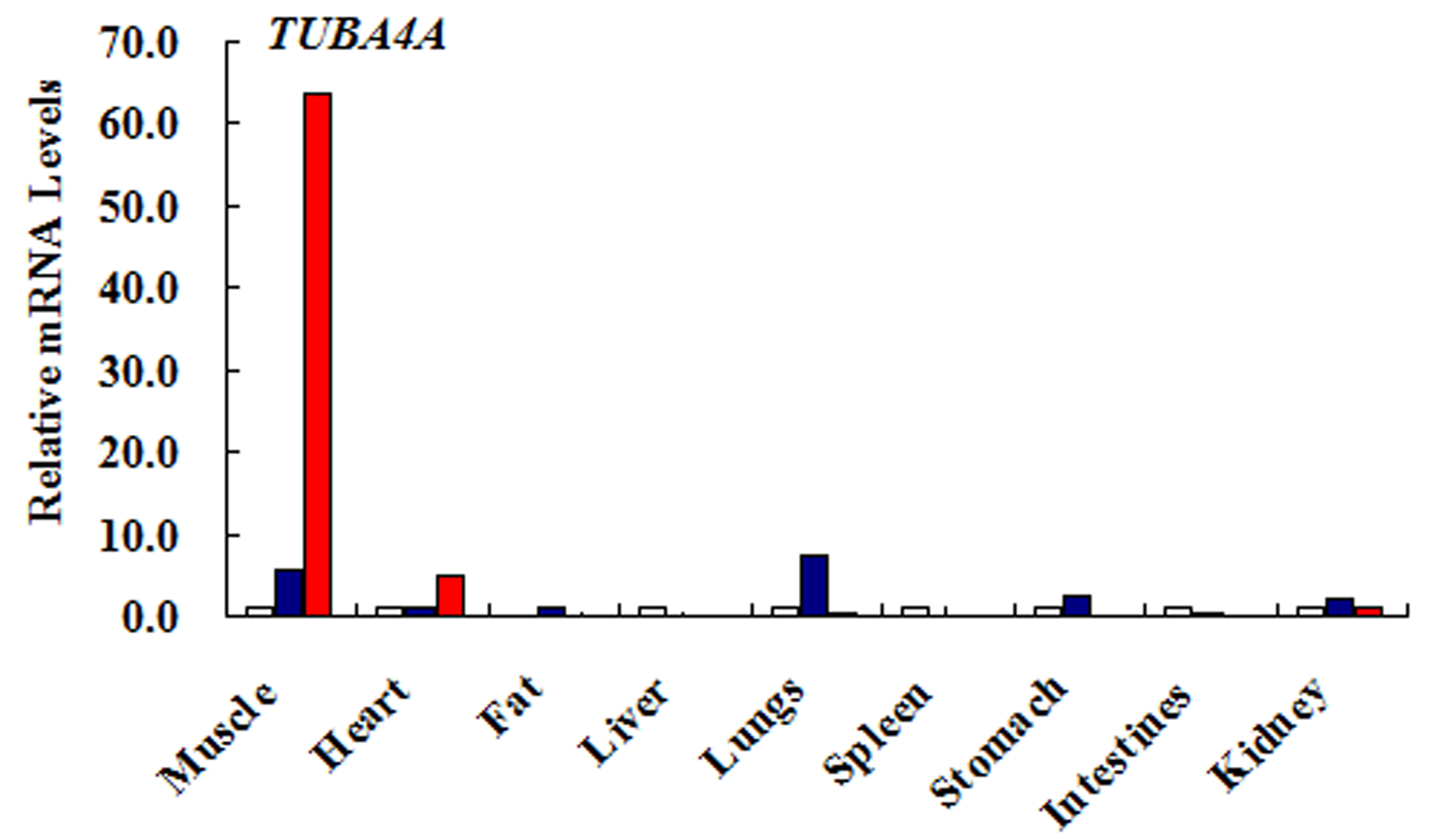

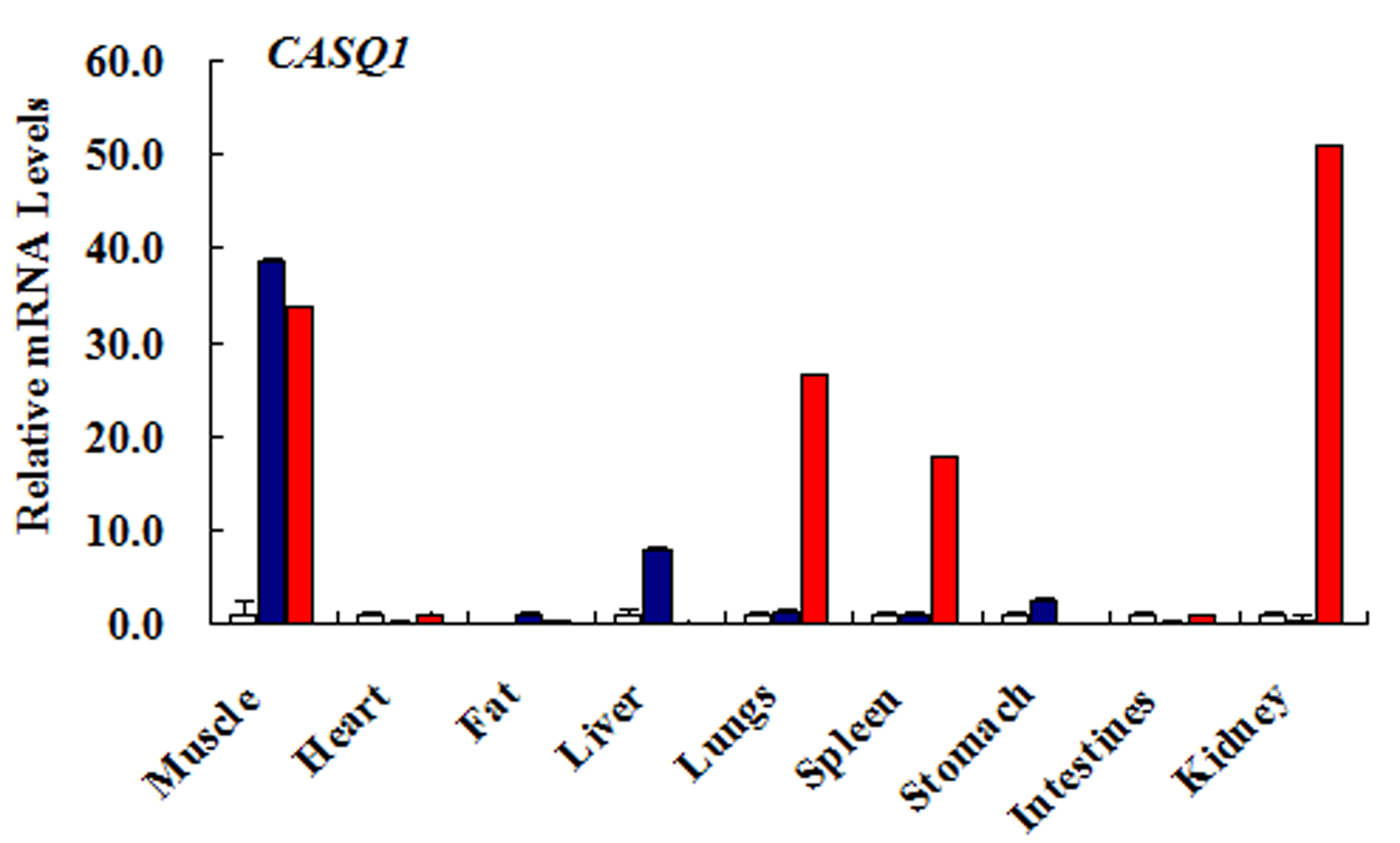


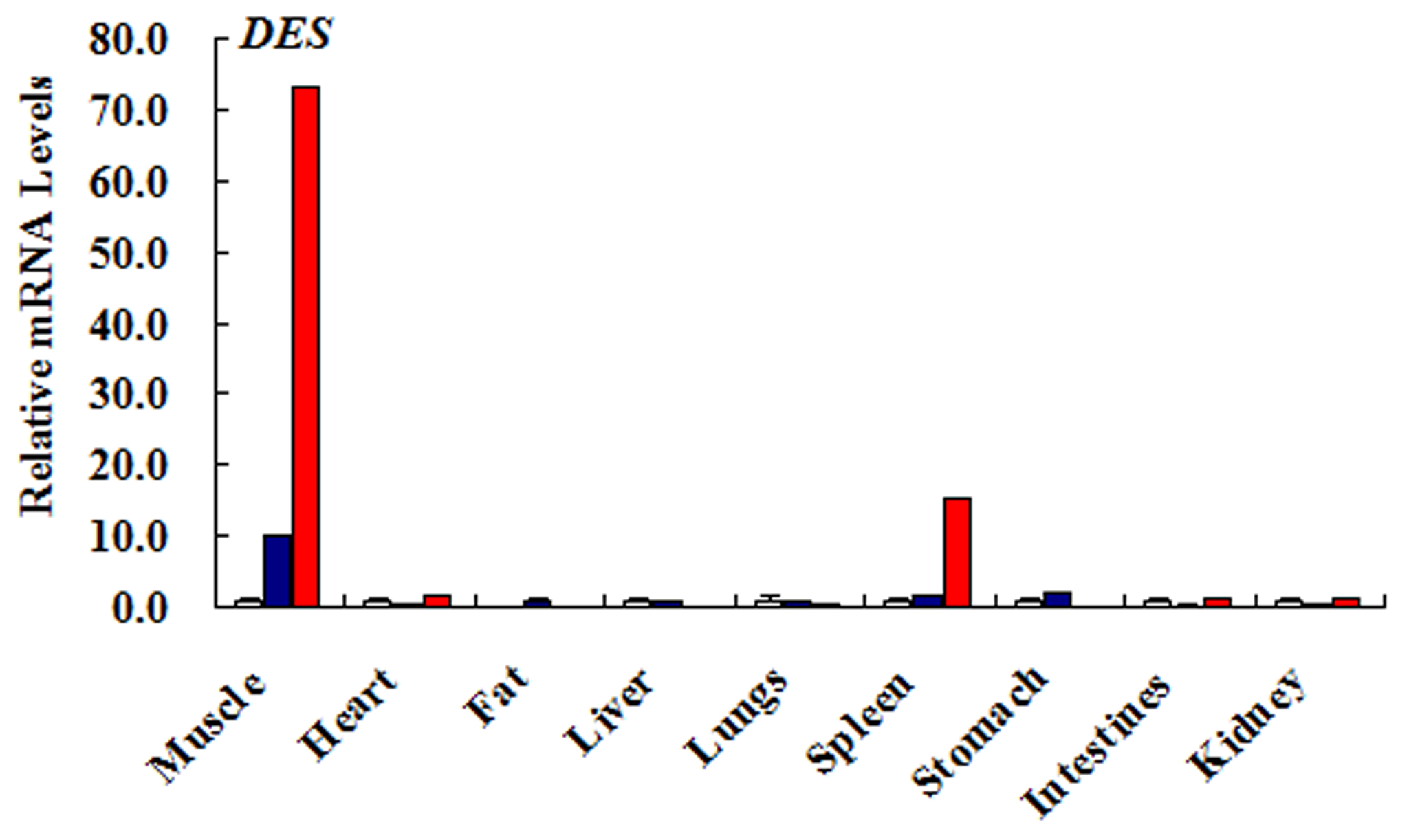

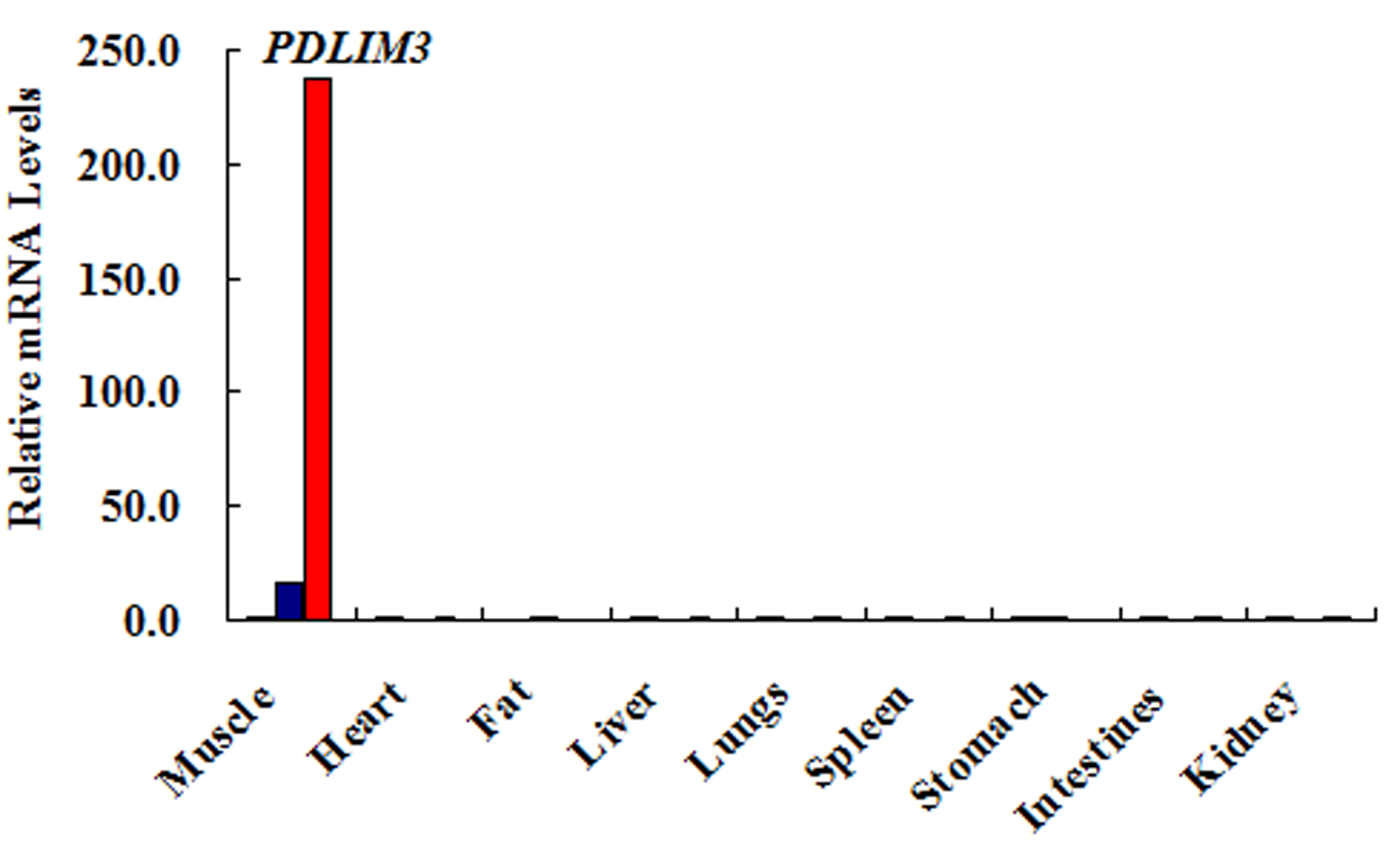


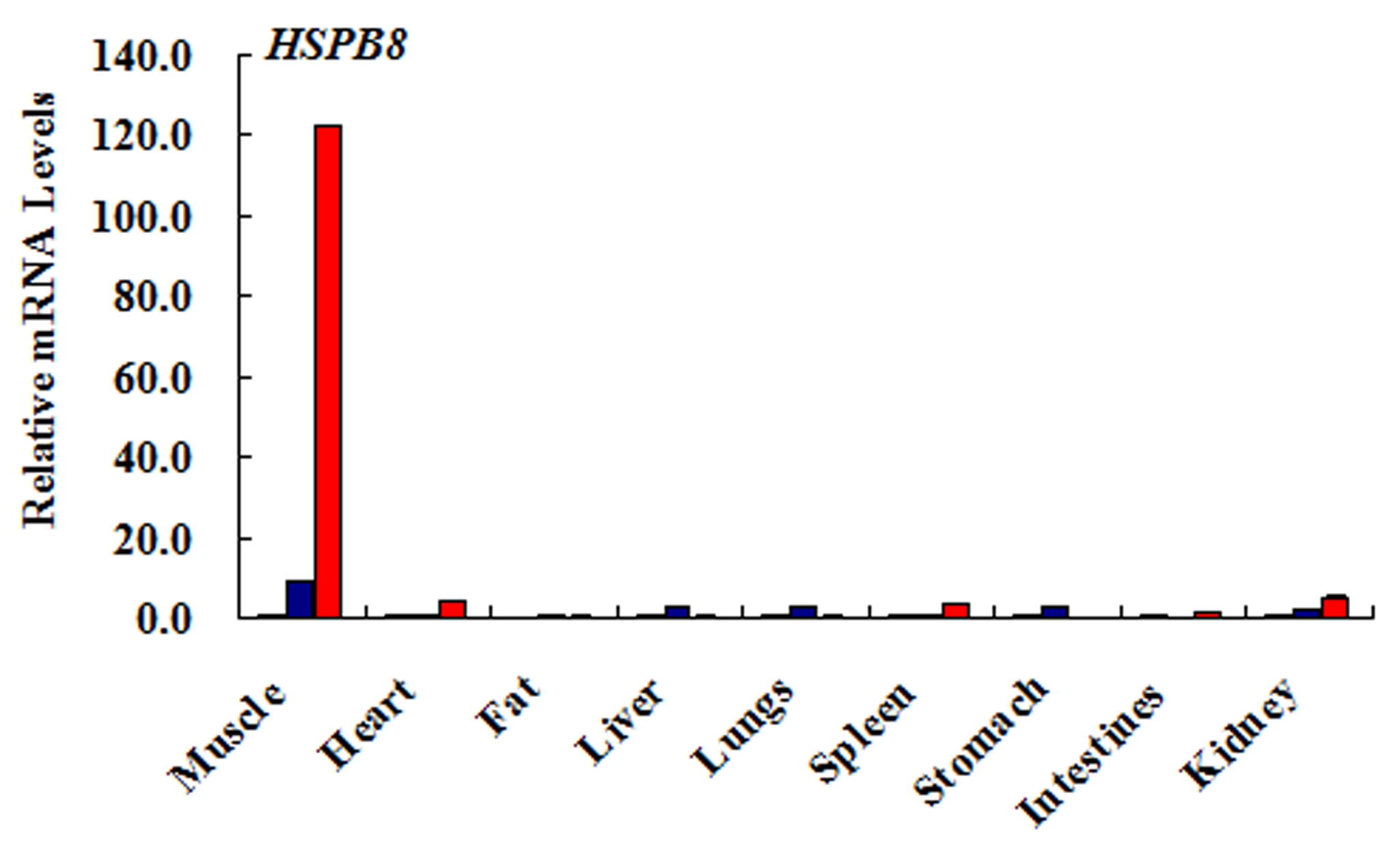

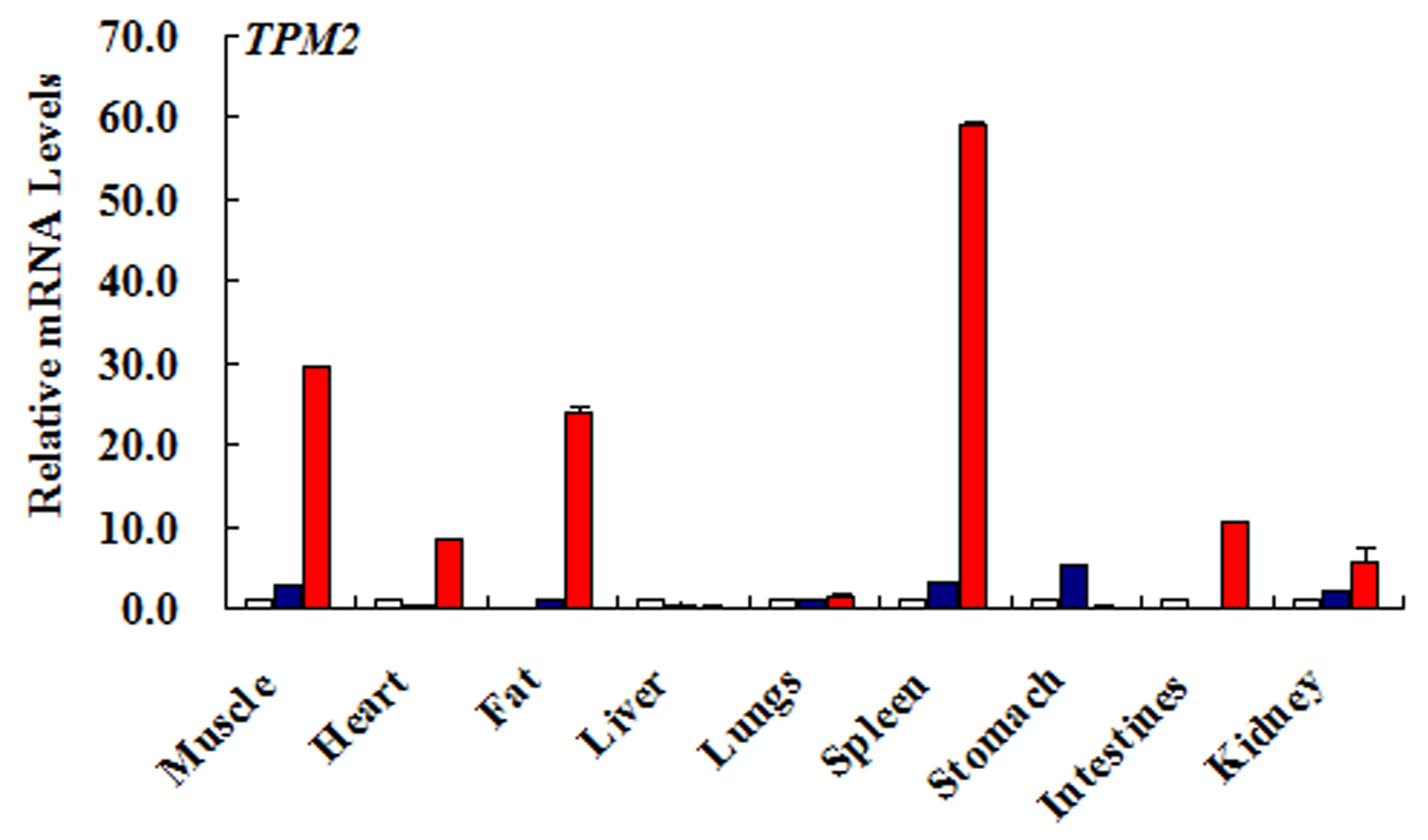


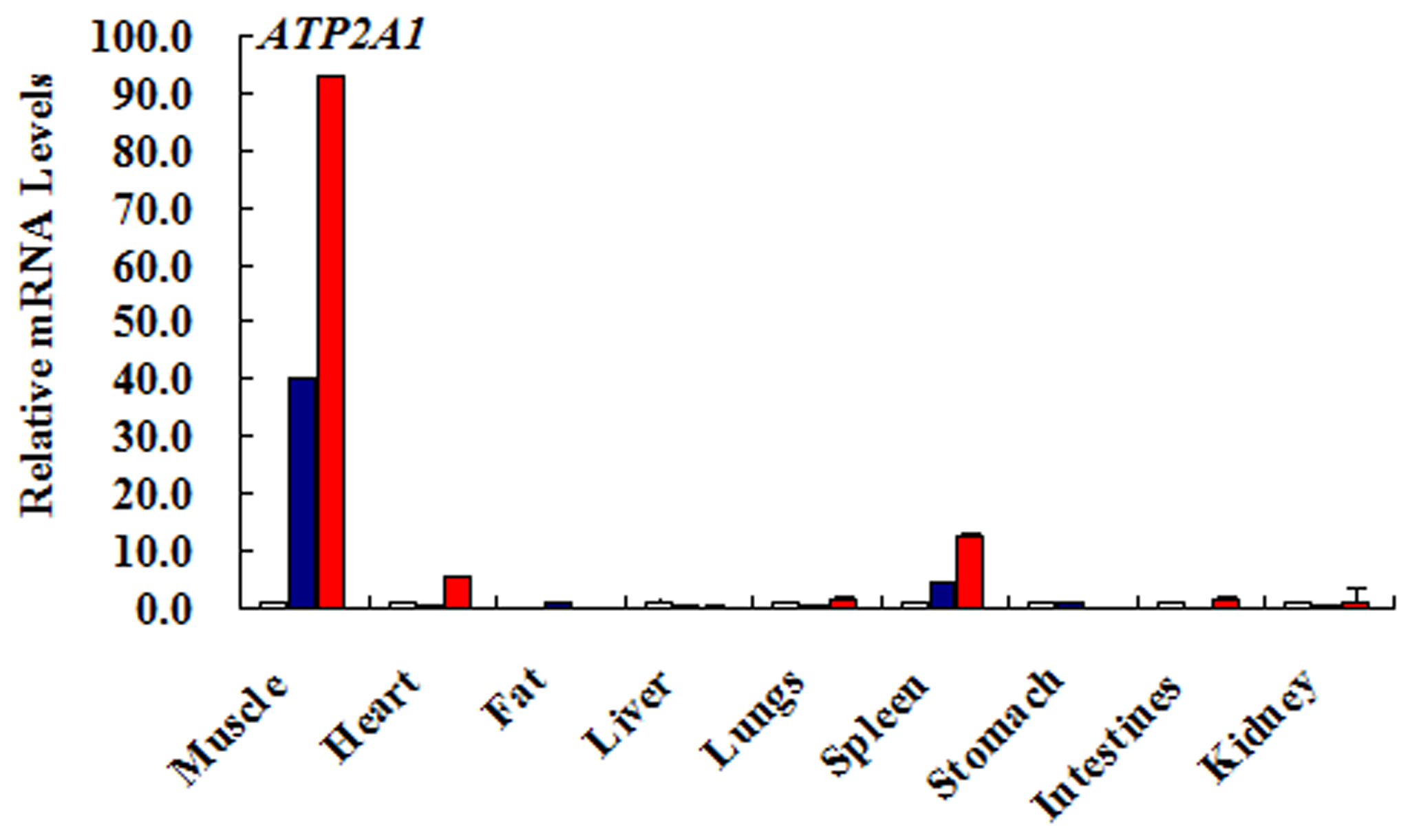

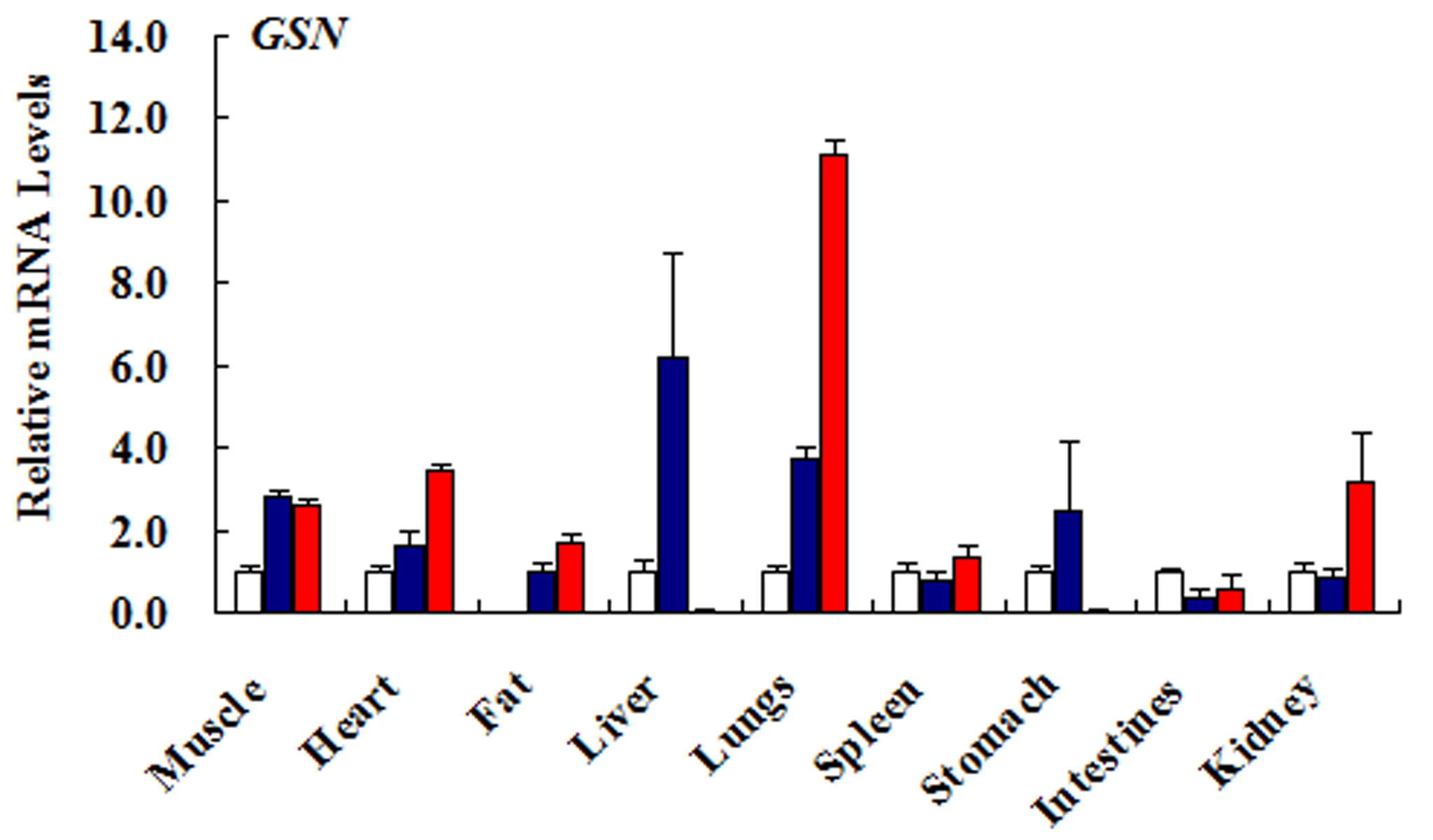


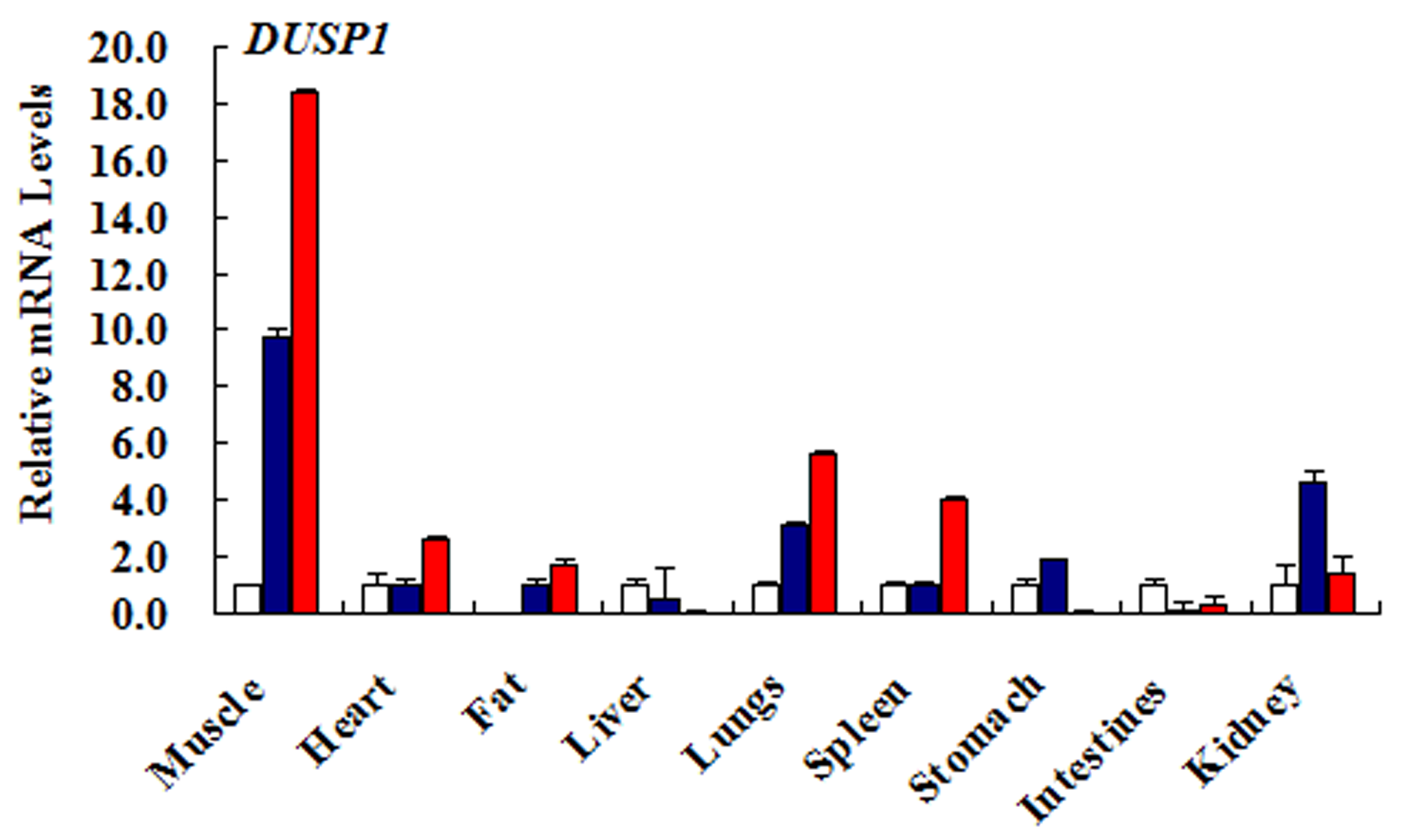

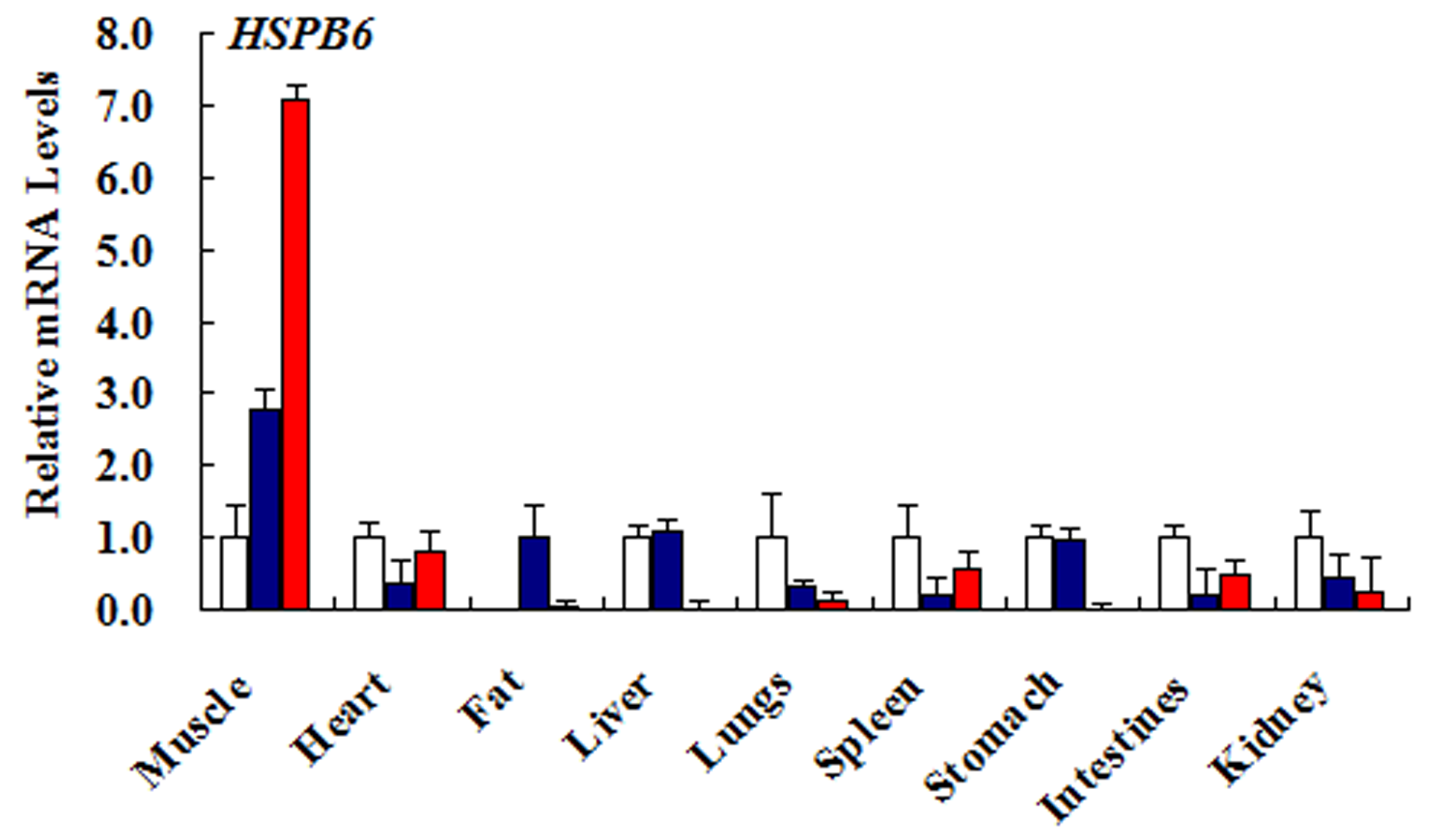


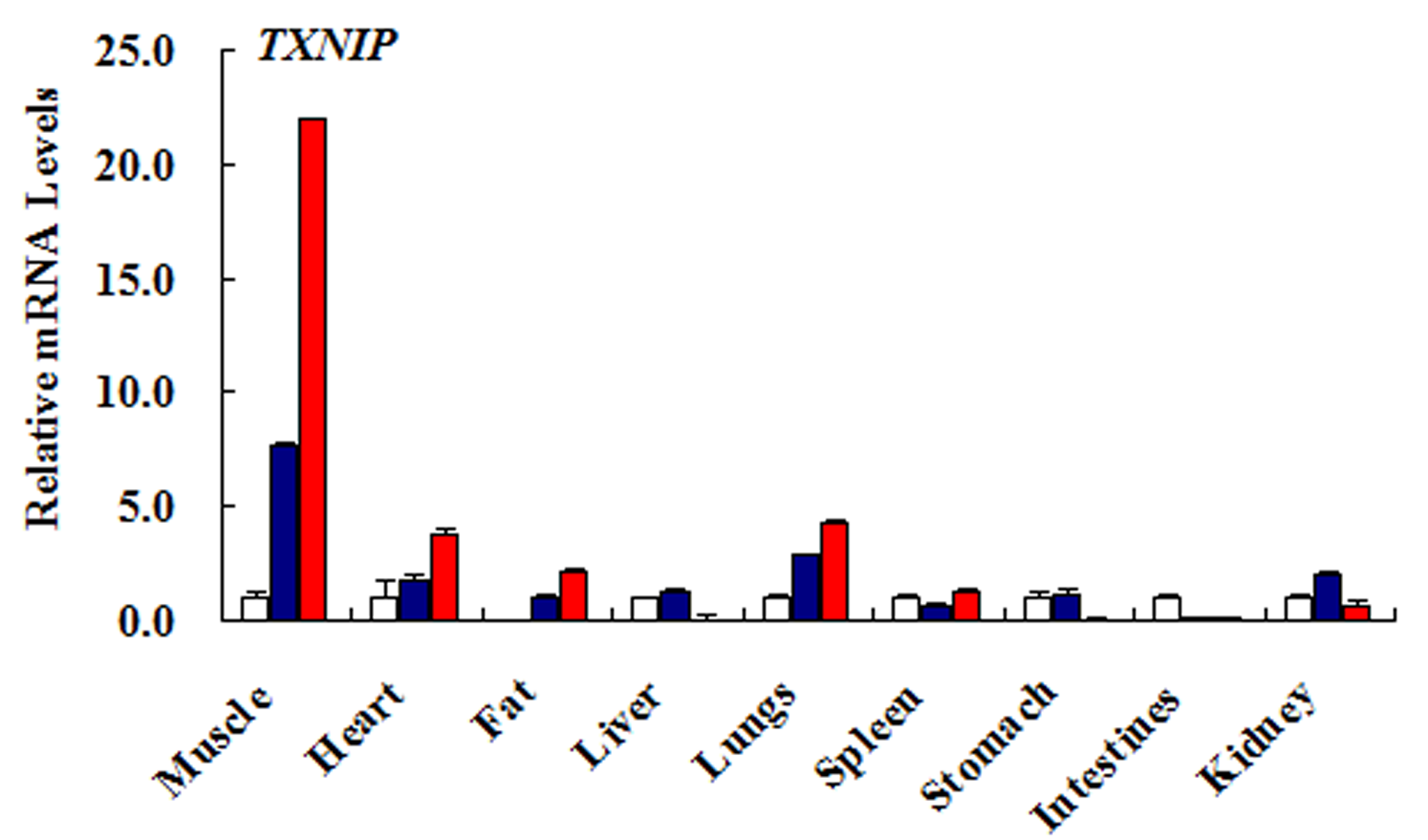

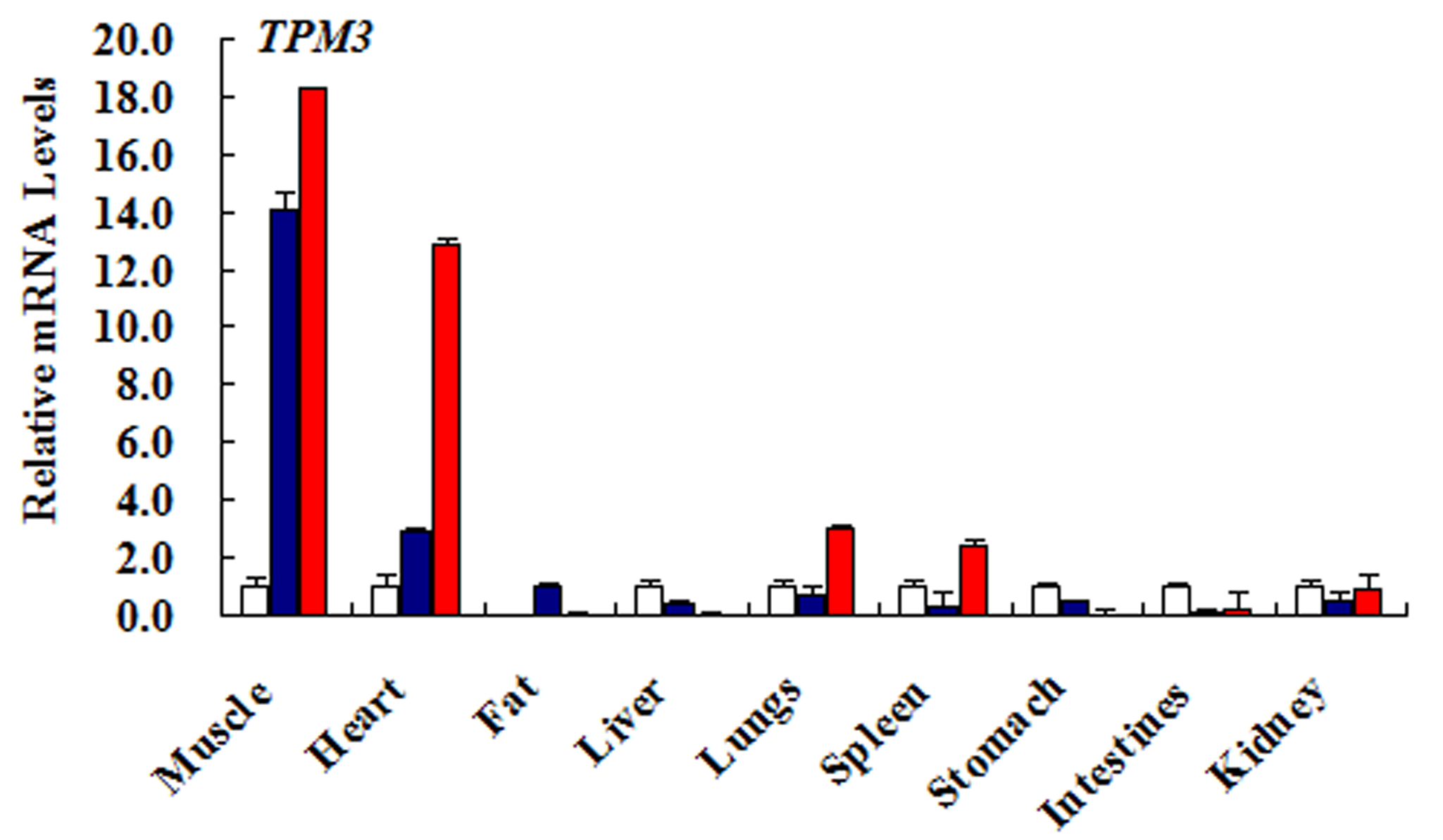


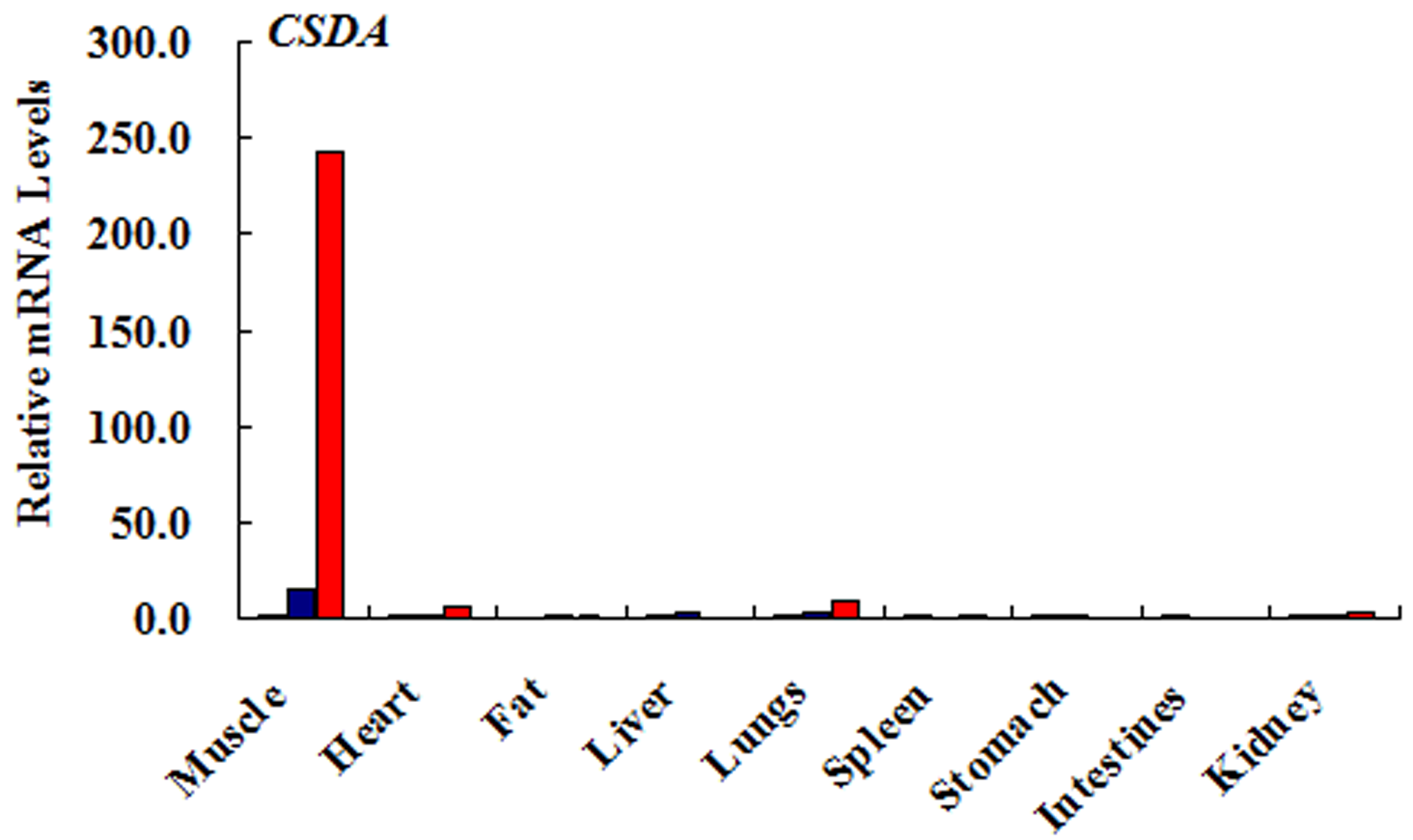

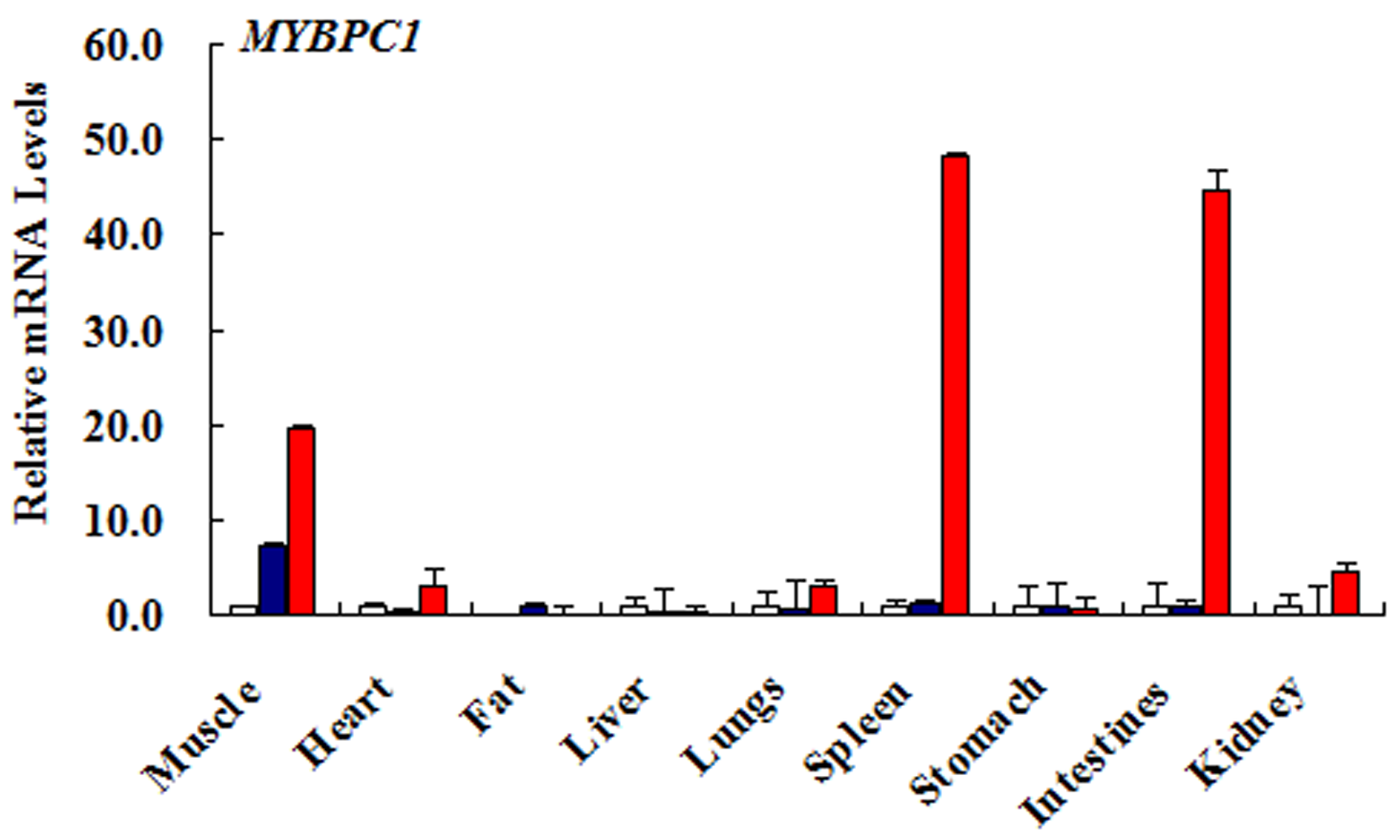


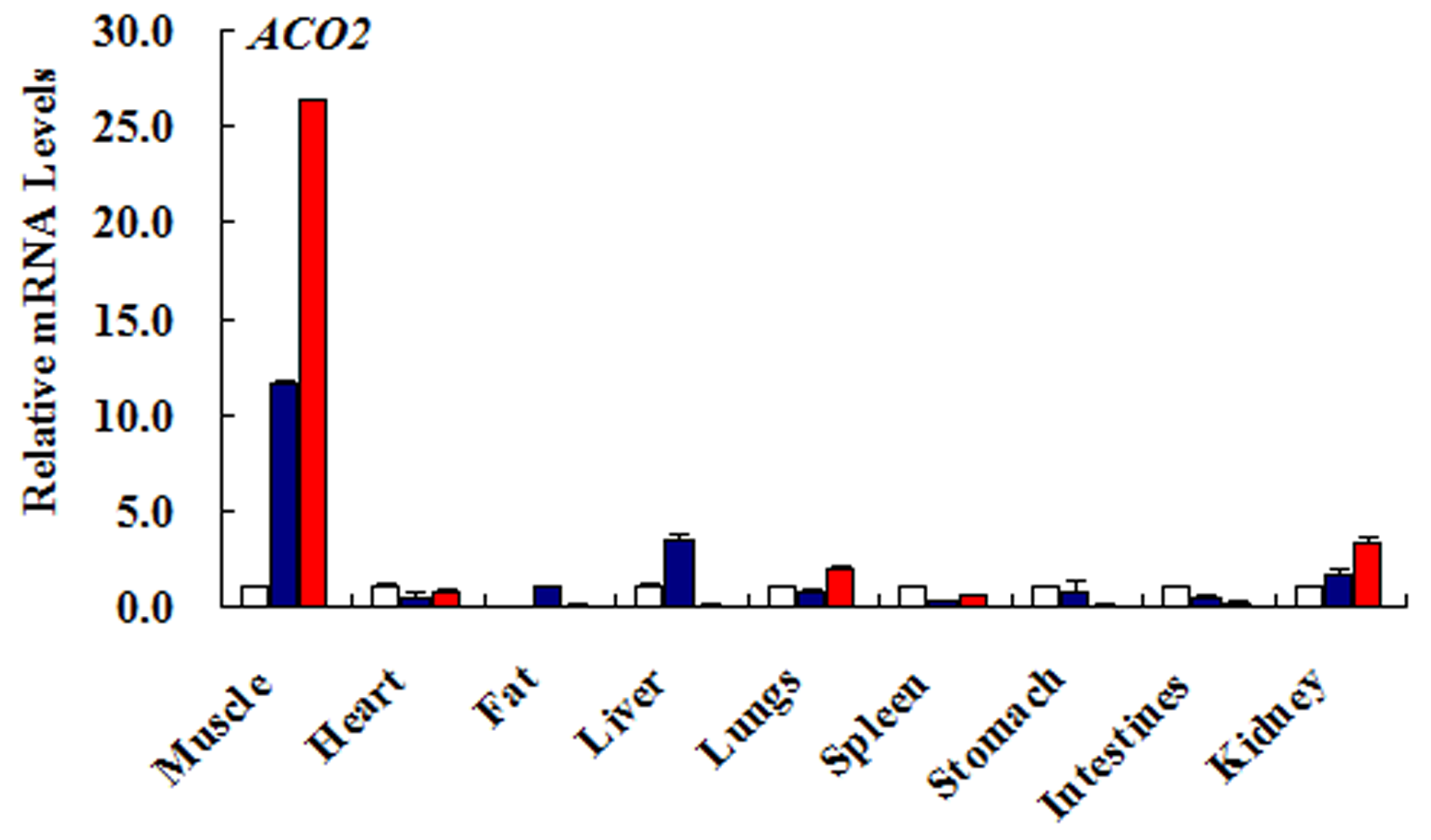

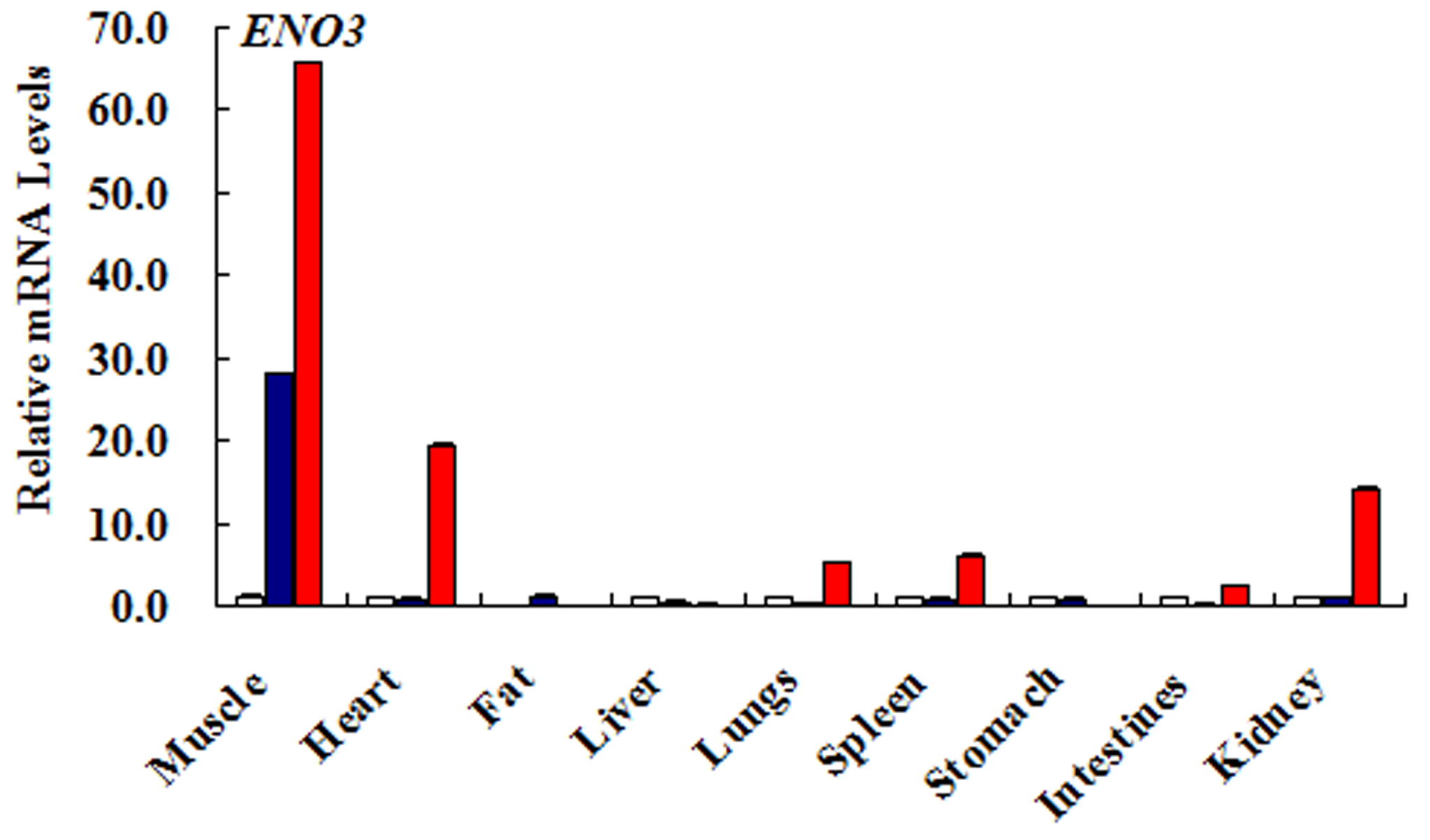


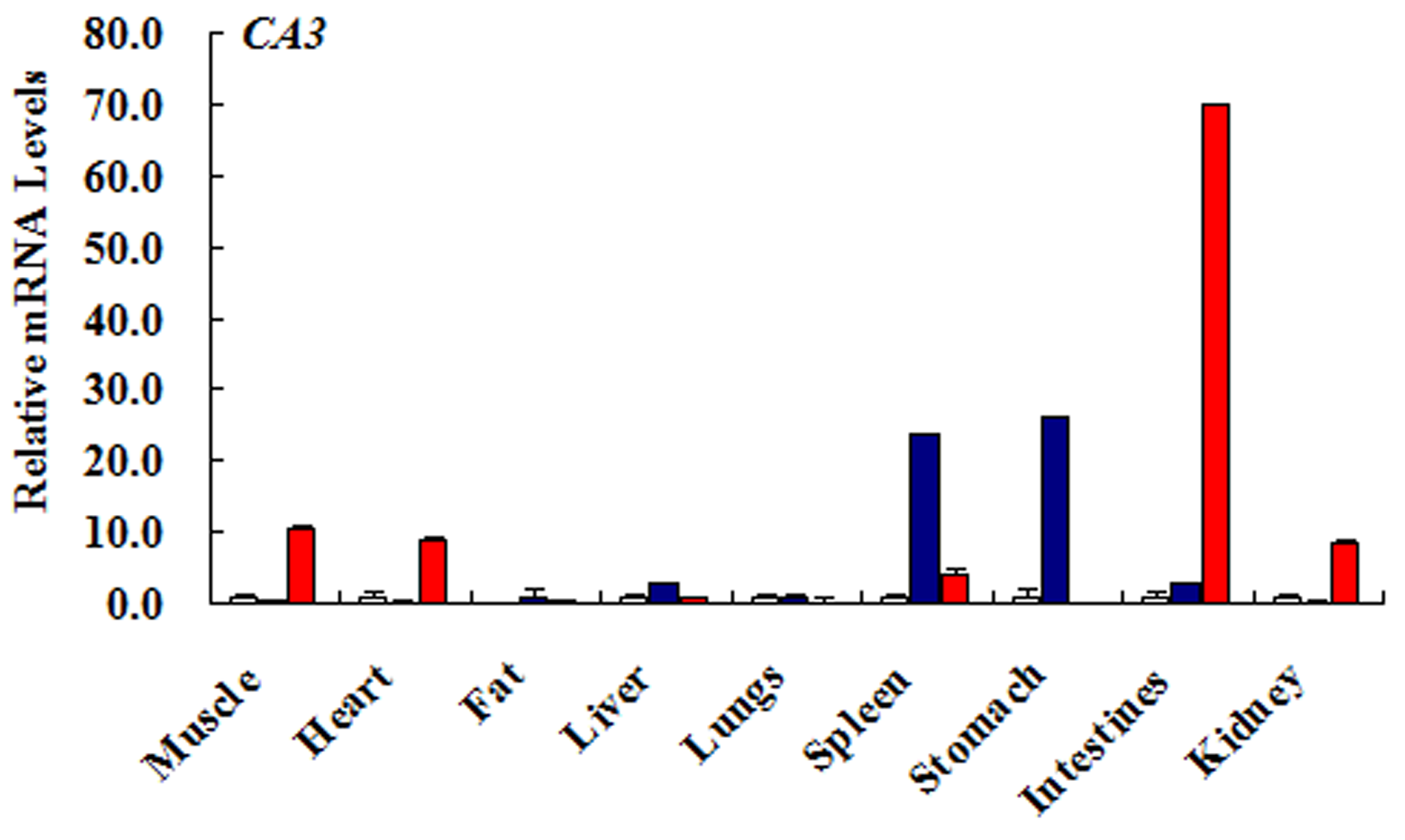

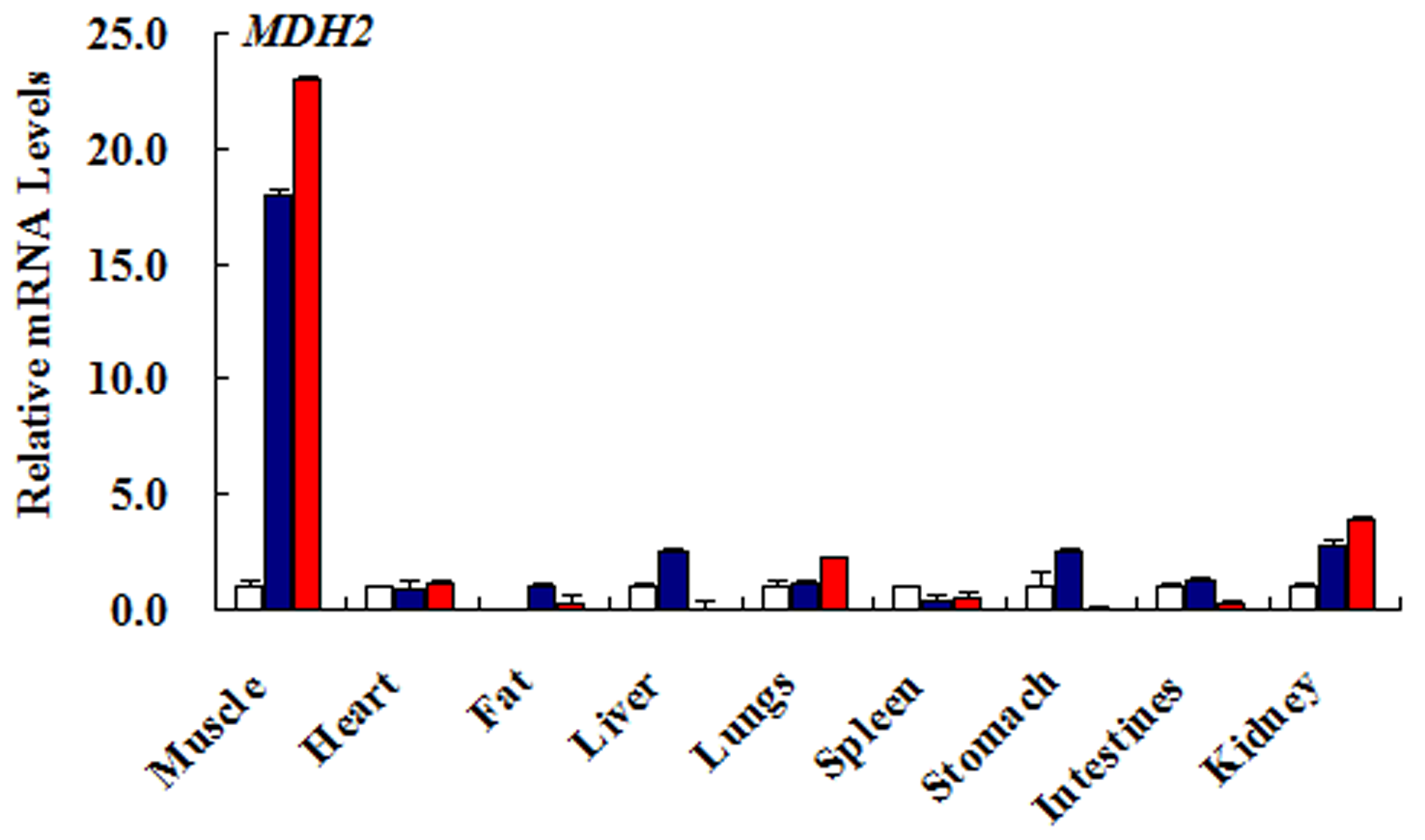


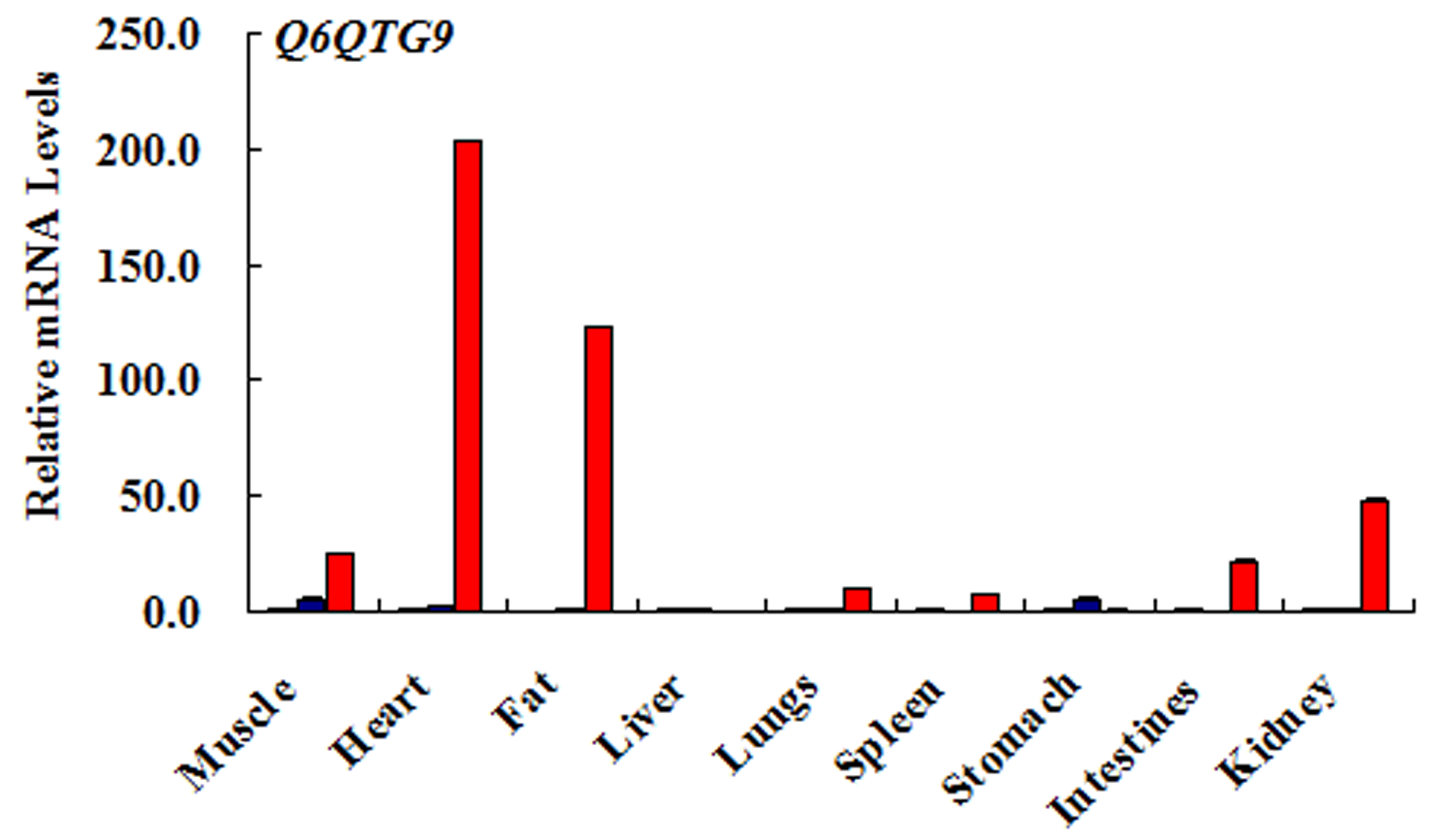

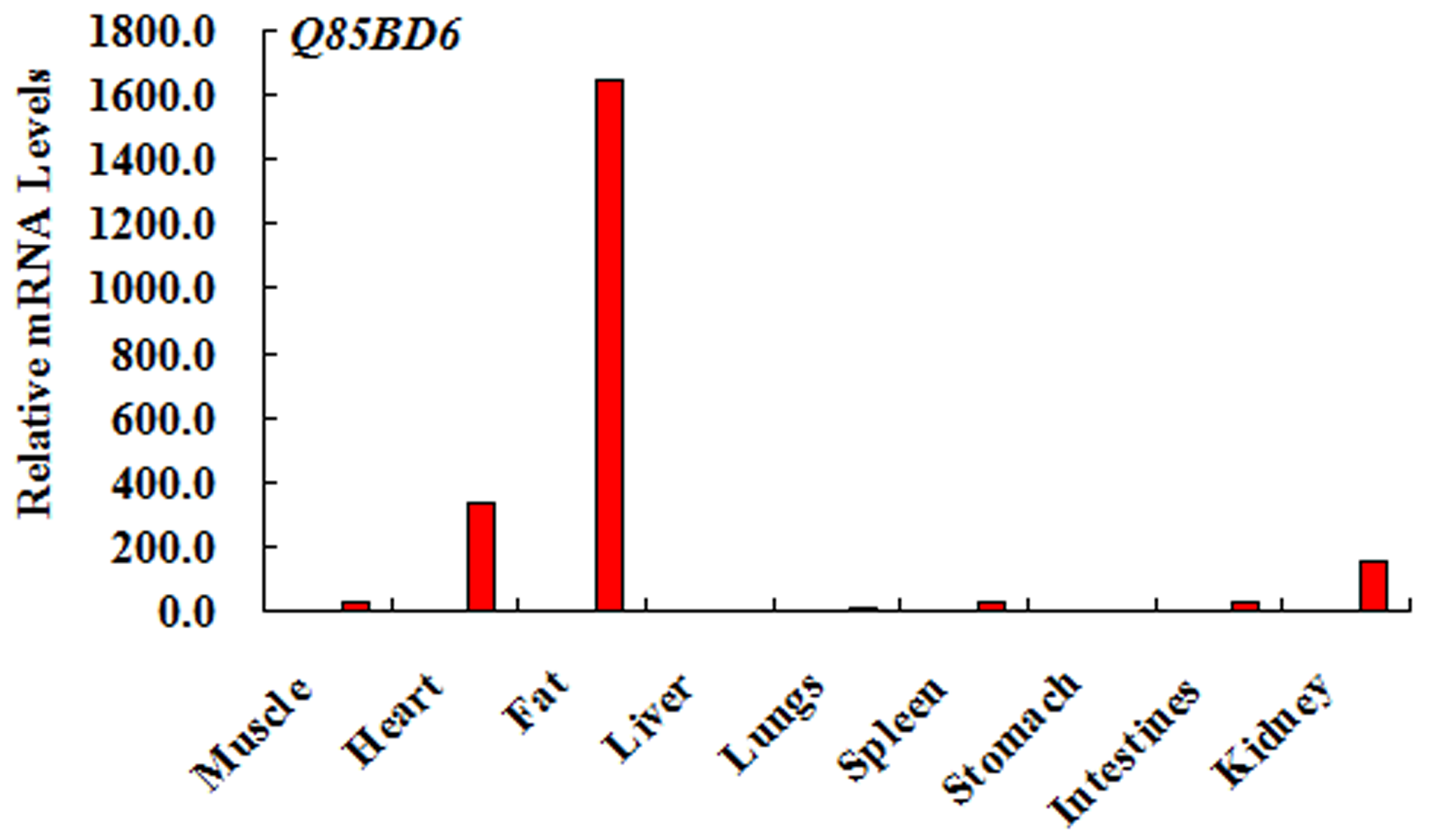


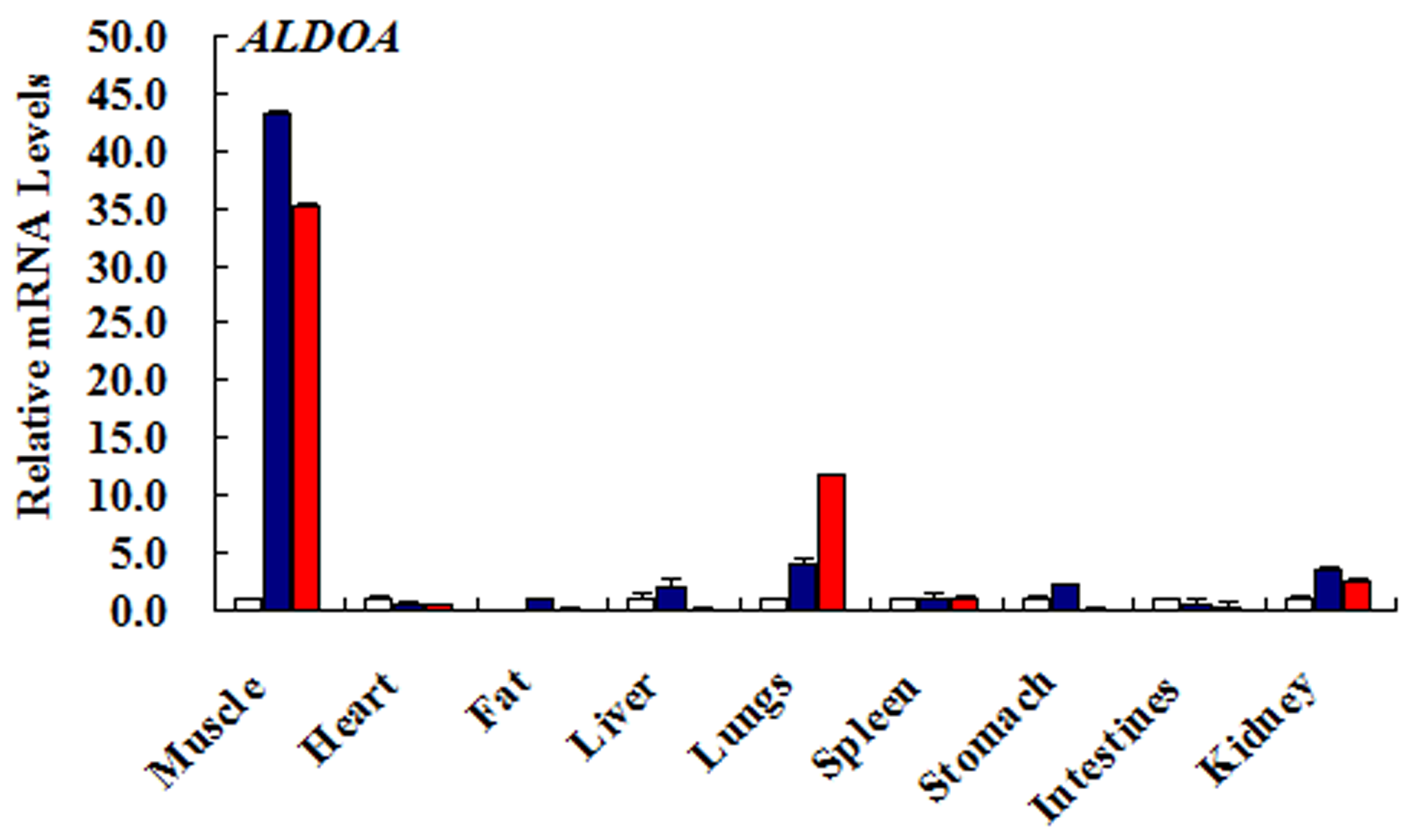

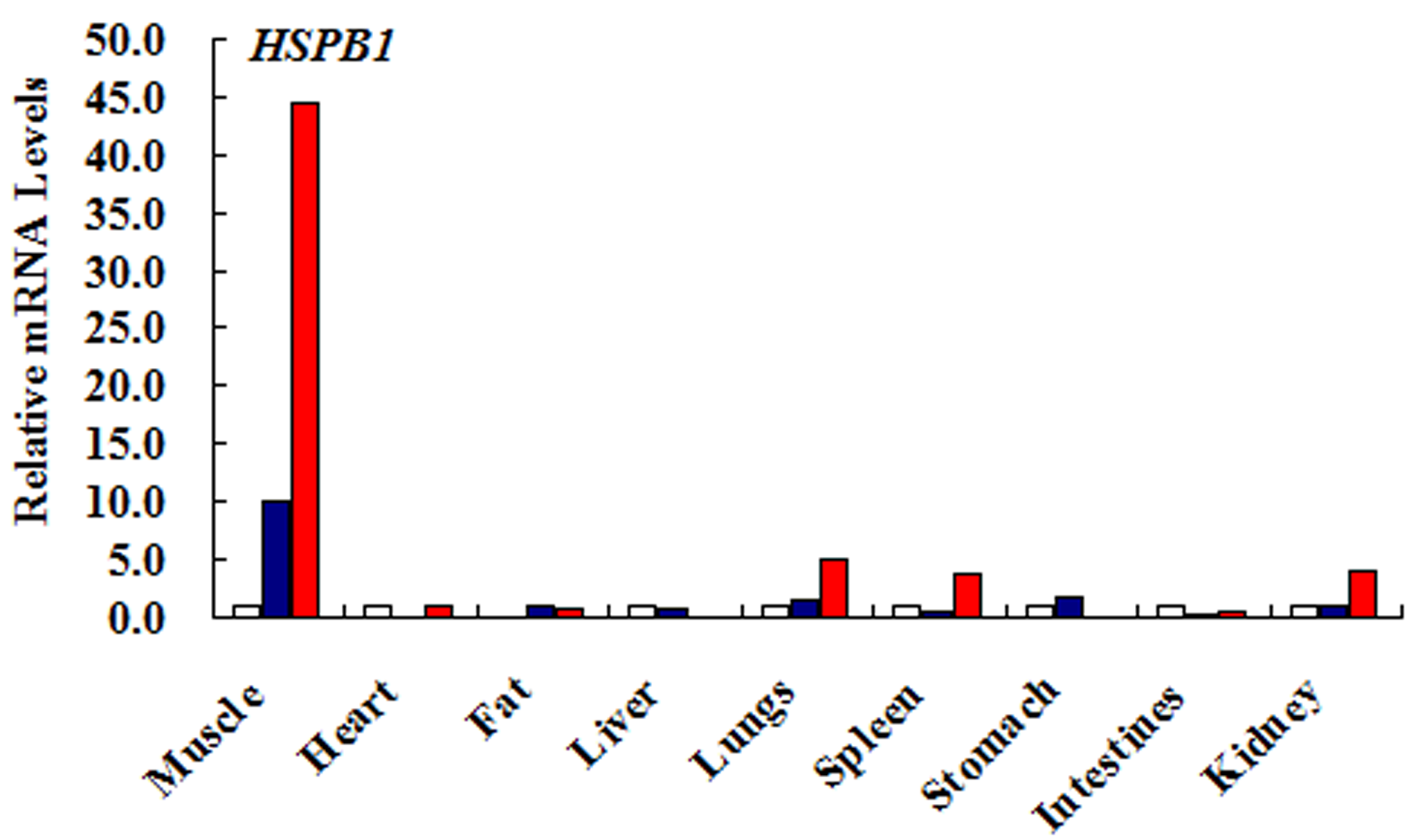


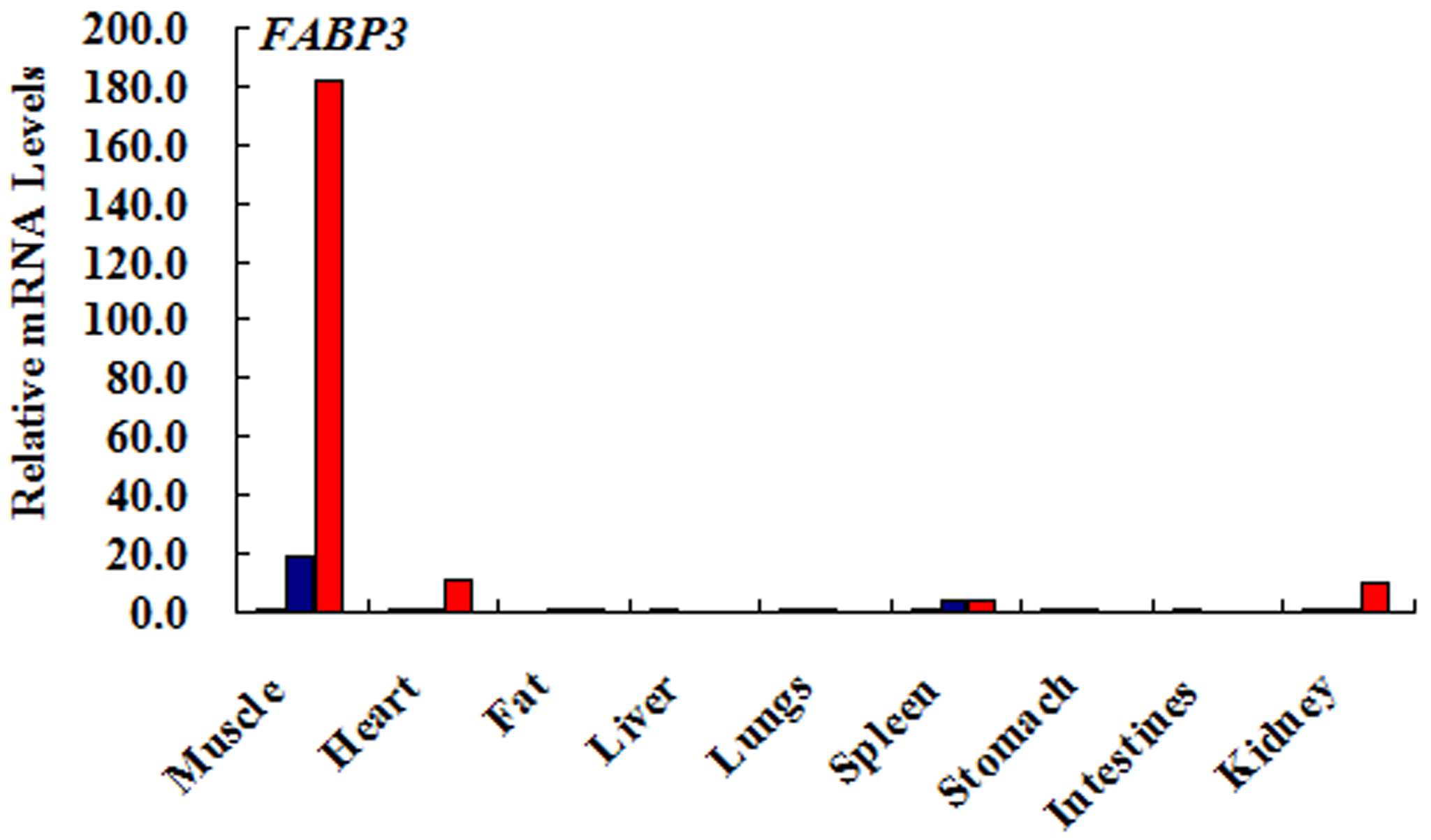

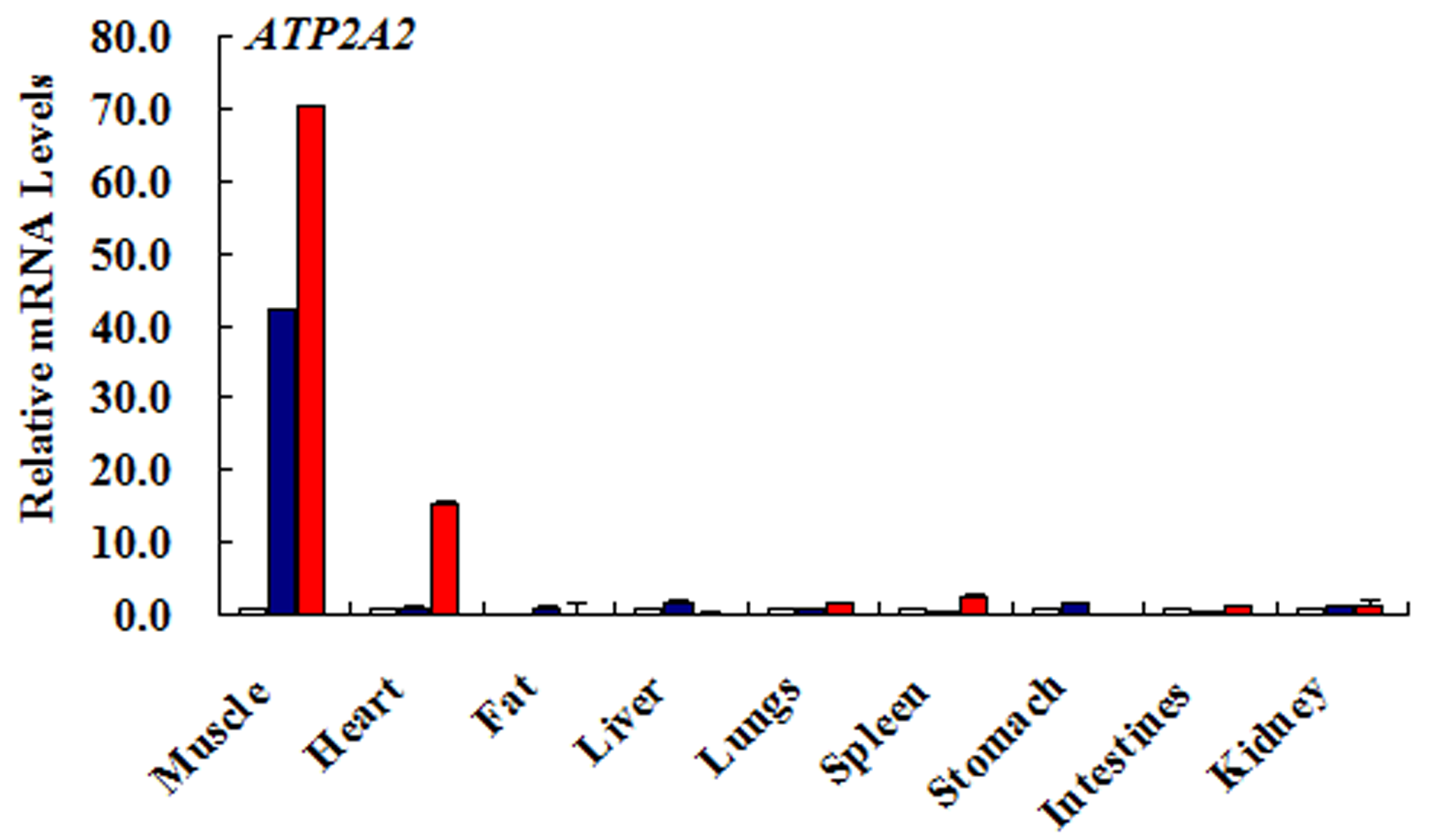


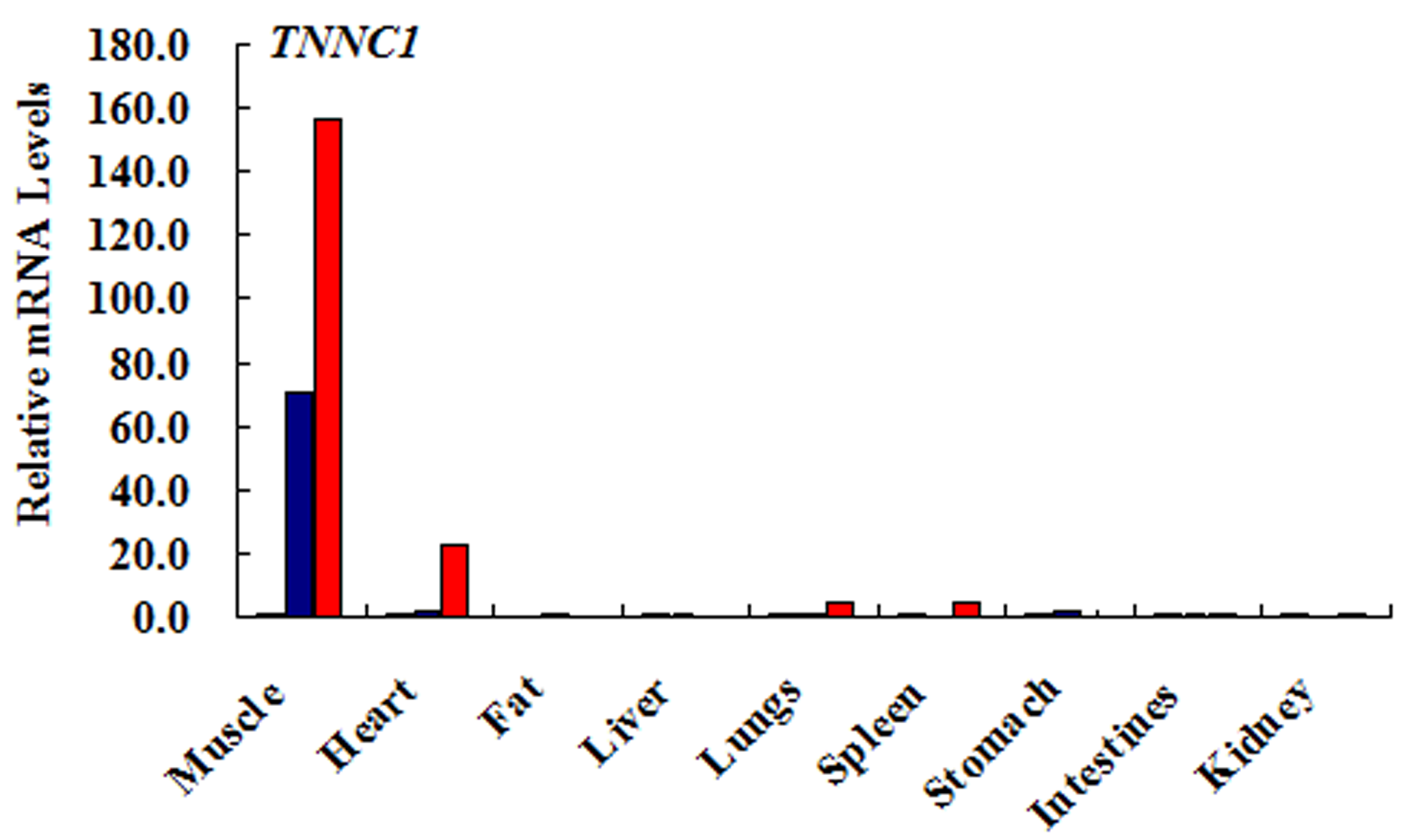
**
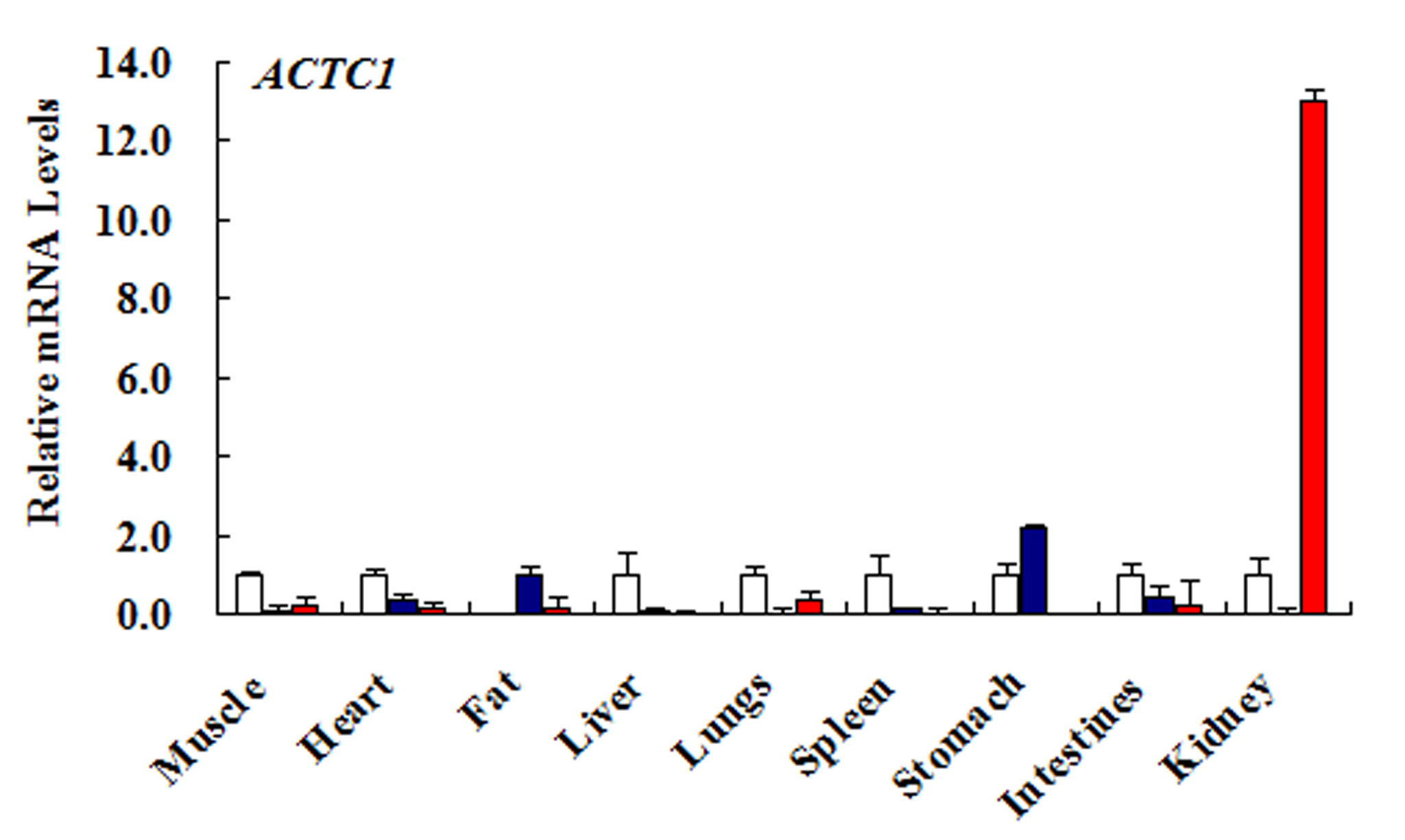
**

**
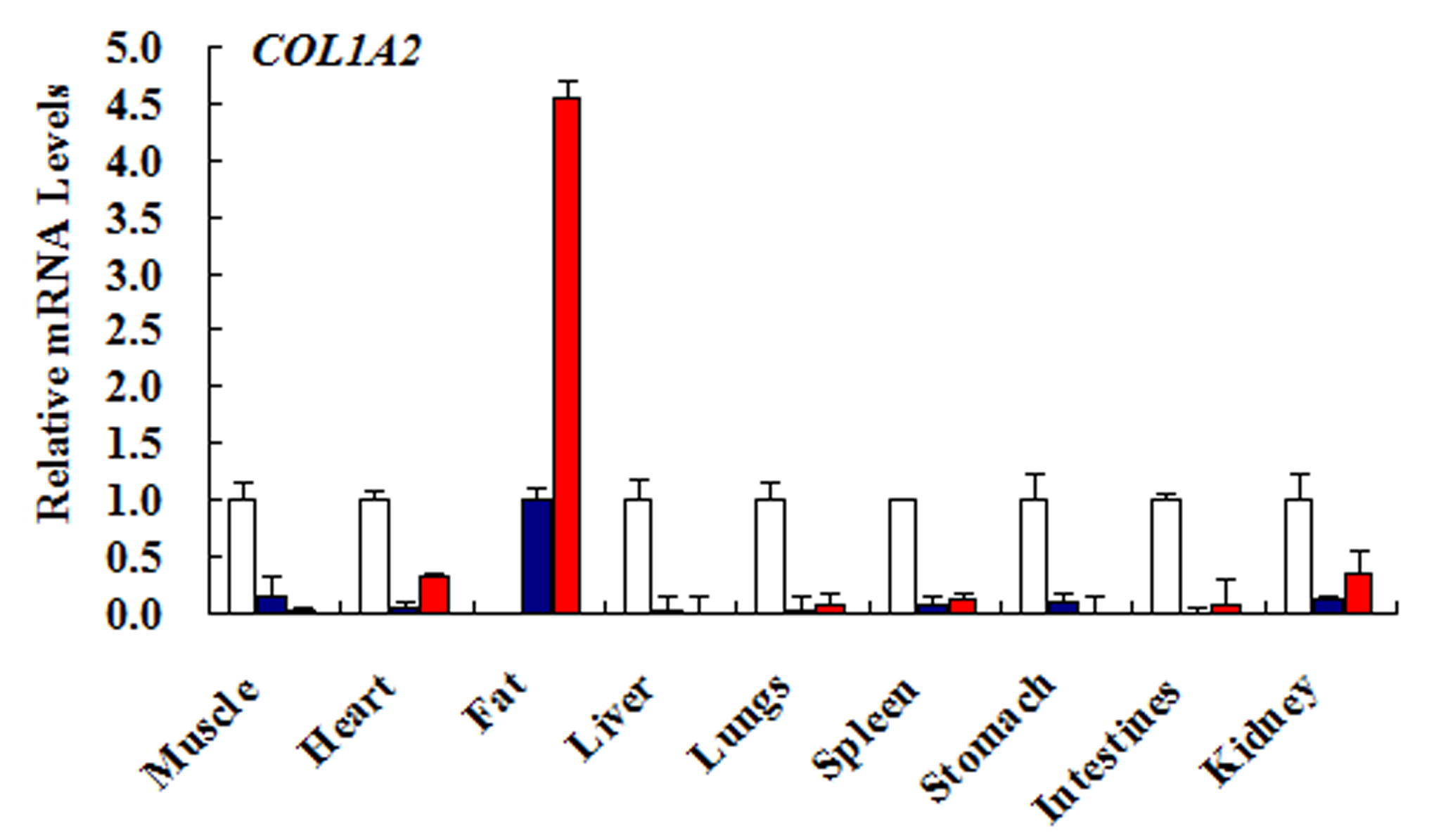

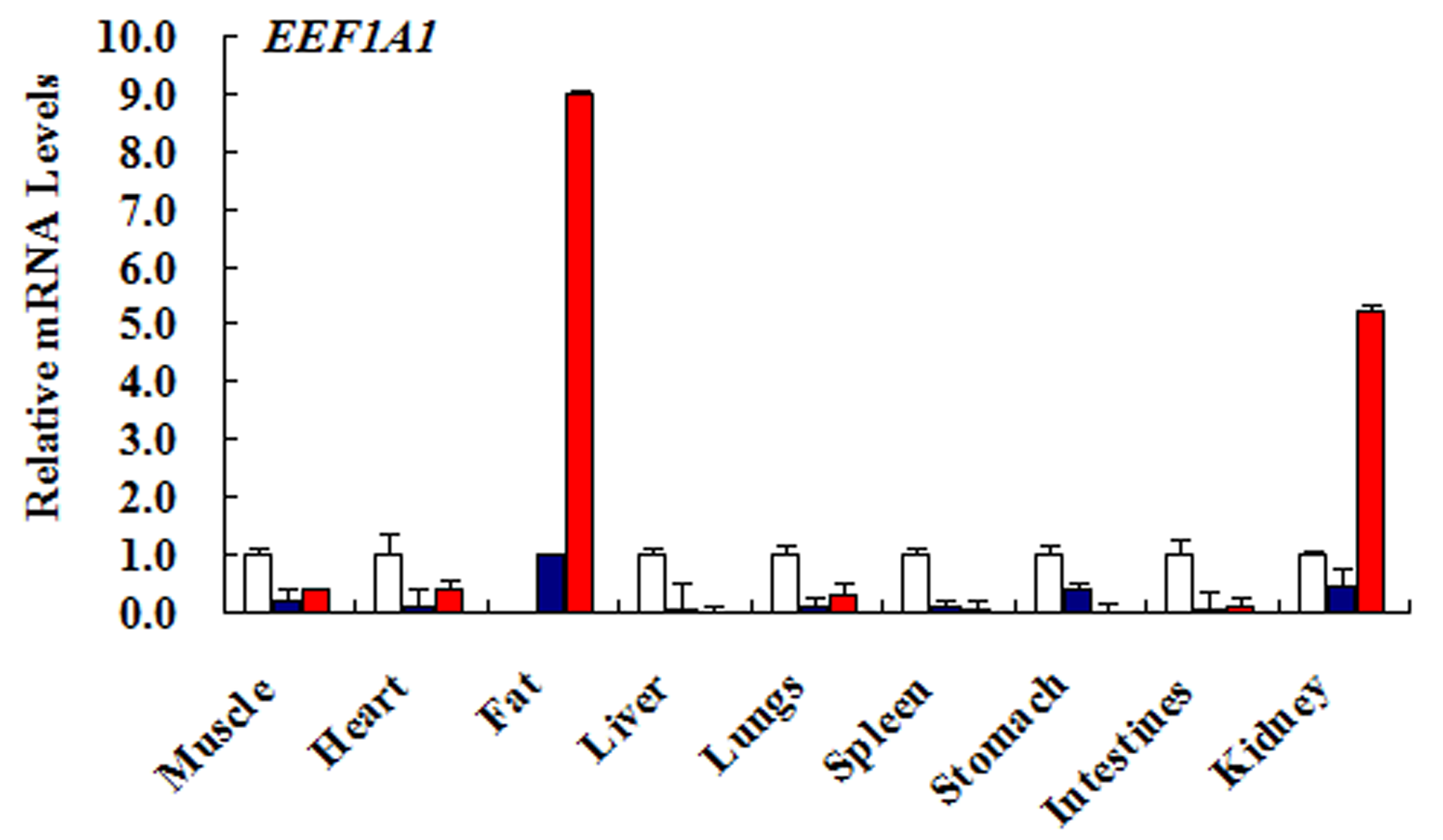
**

**
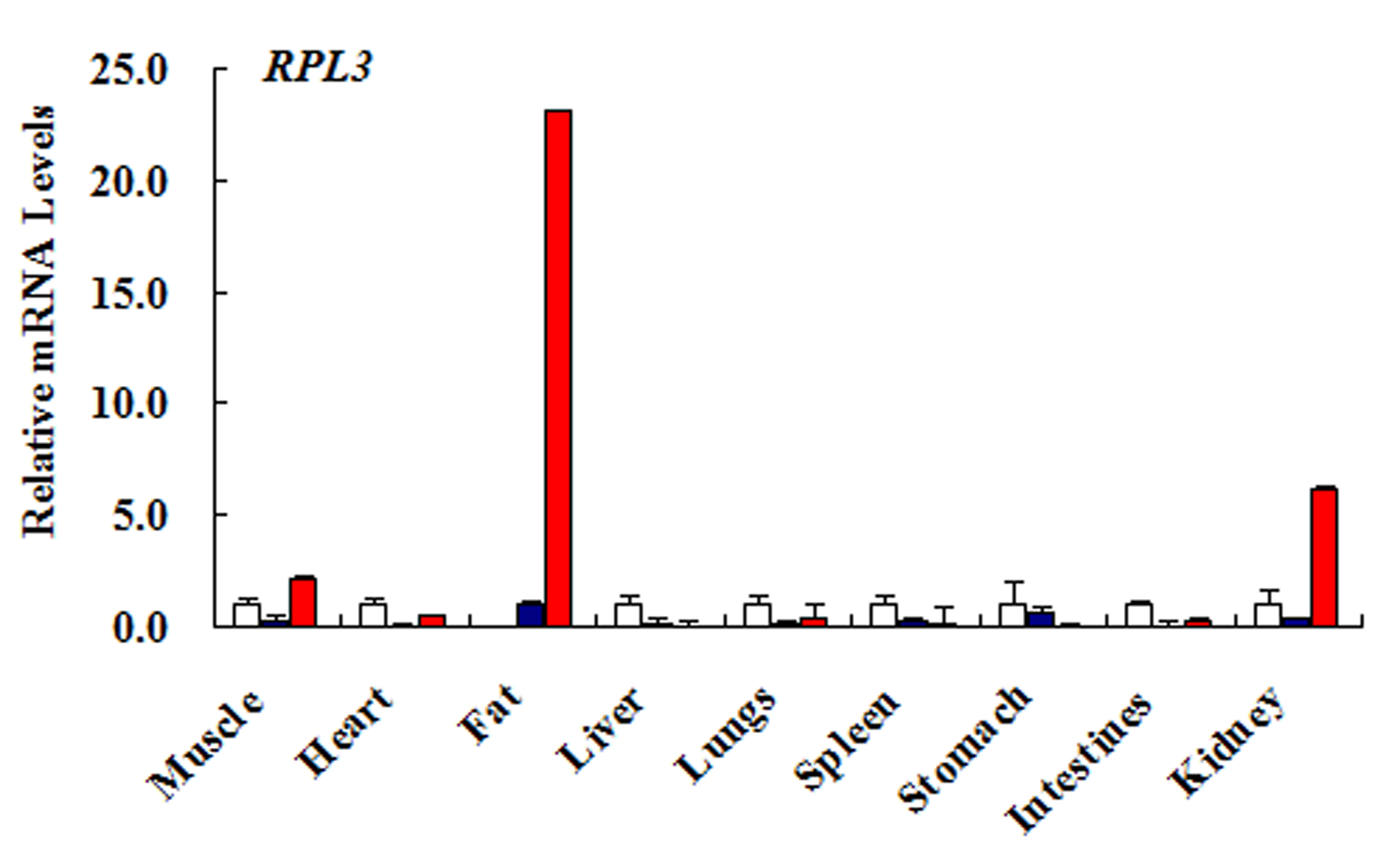

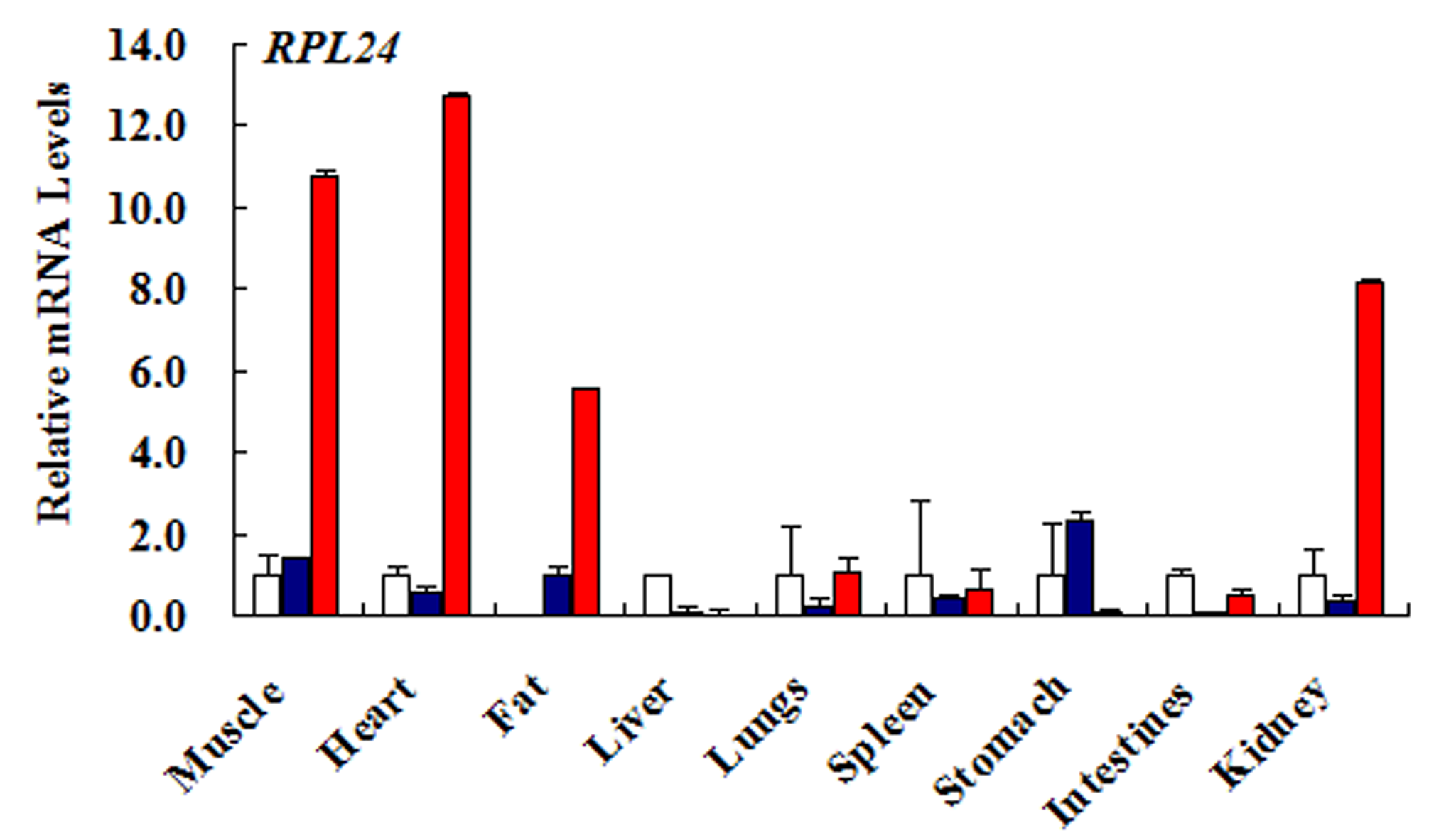
**

**
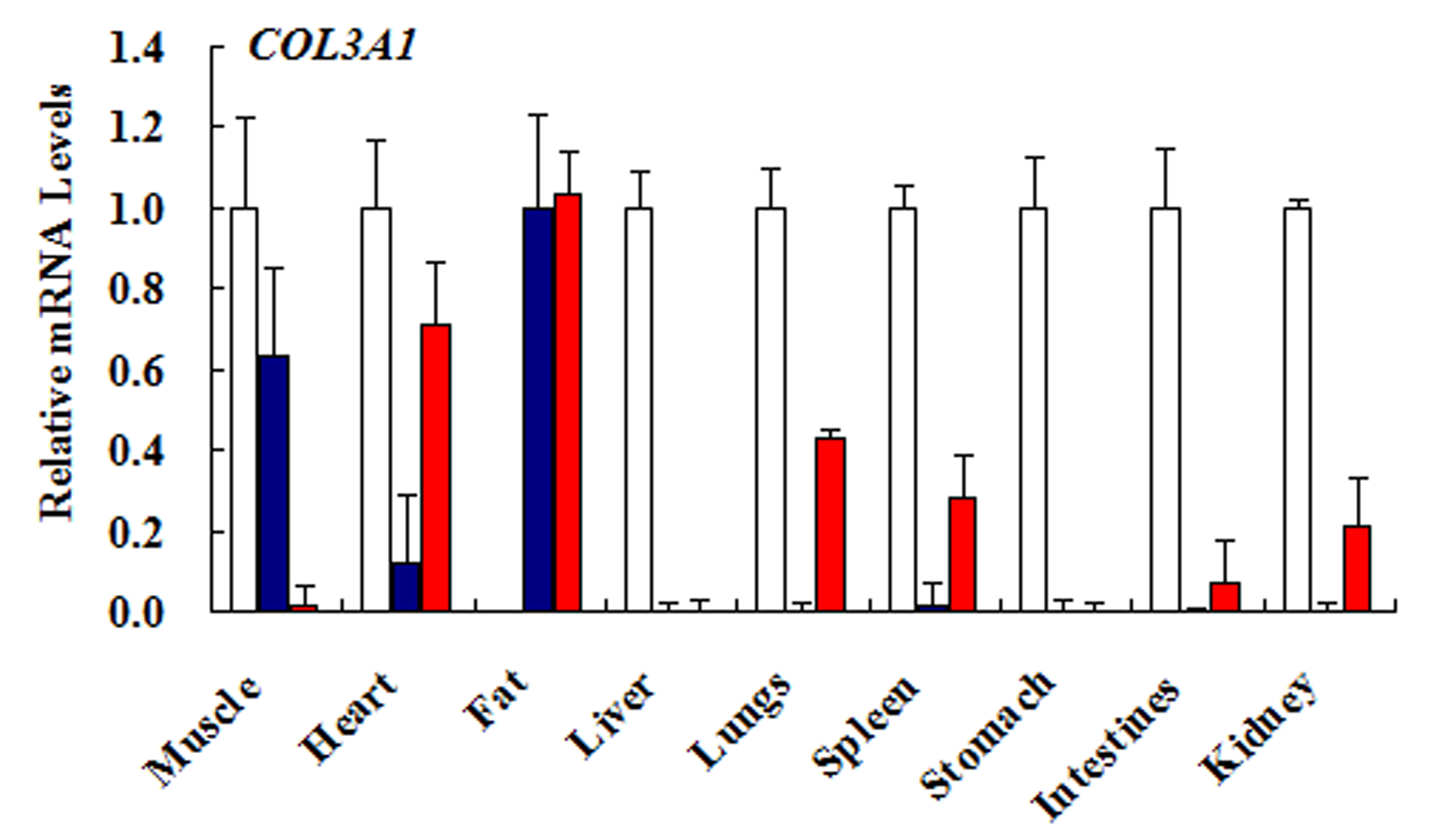

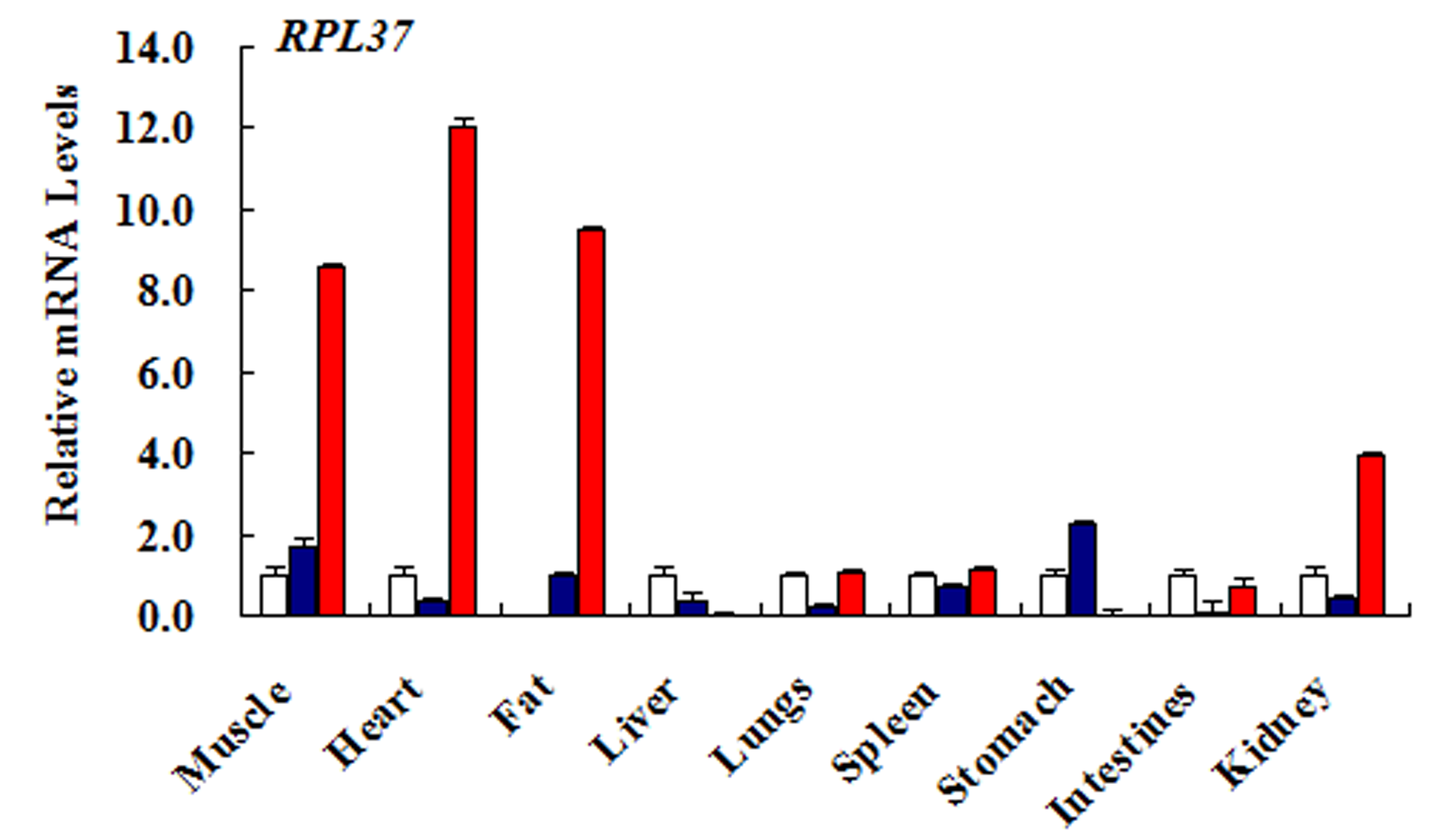
**

**
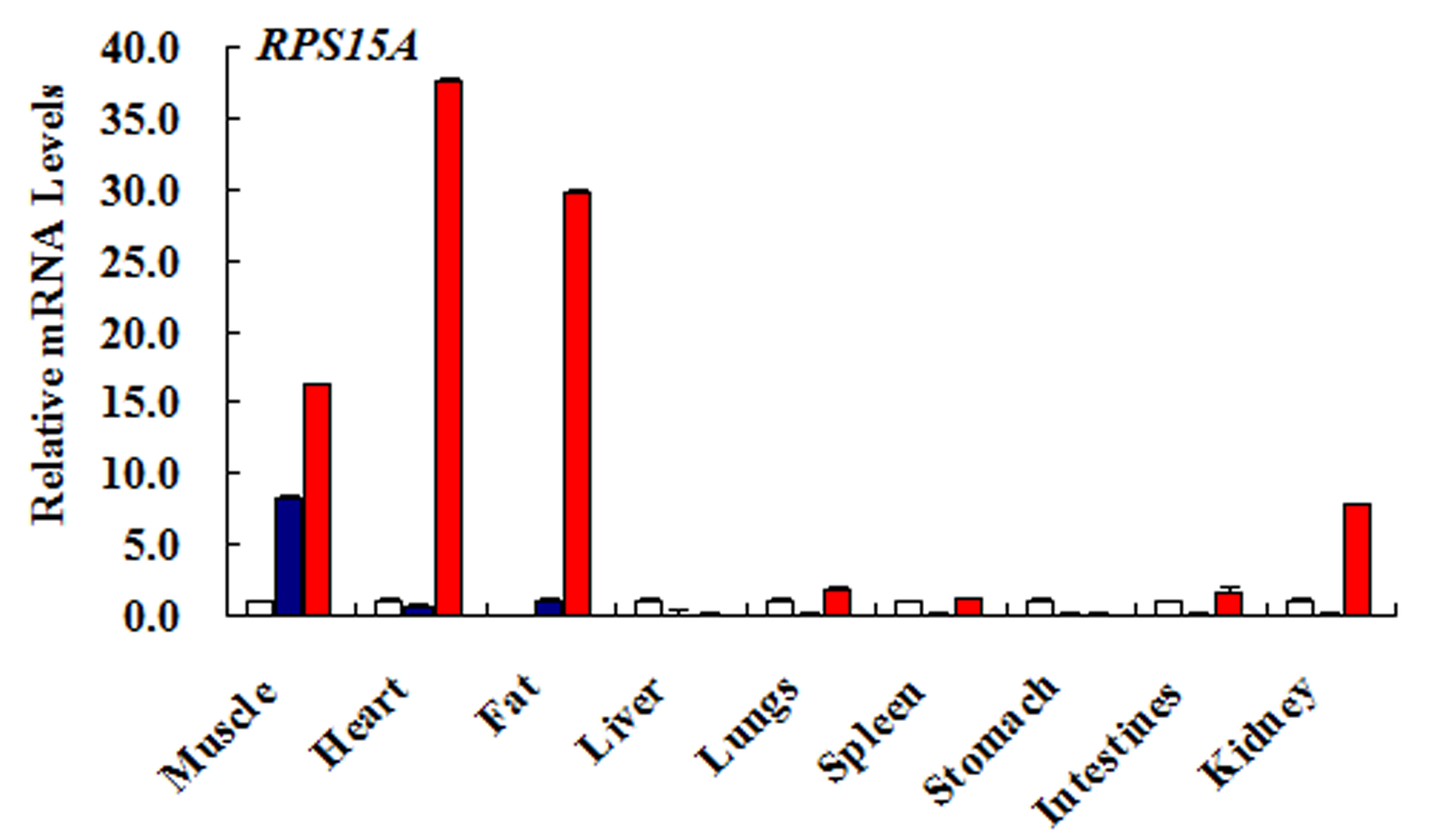
**
